# Heterokaryon-Based Reprogramming of Human B Lymphocytes for Pluripotency Requires Oct4 but Not Sox2

**DOI:** 10.1371/journal.pgen.1000170

**Published:** 2008-09-05

**Authors:** Carlos F. Pereira, Rémi Terranova, Natalie K. Ryan, Joana Santos, Kelly J. Morris, Wei Cui, Matthias Merkenschlager, Amanda G. Fisher

**Affiliations:** 1 Lymphocyte Development Group, MRC Clinical Sciences Centre, Imperial College School of Medicine, Hammersmith Hospital, London, United Kingdom; 2 Stem Cell Initiative, Institute of Reproductive and Developmental Biology, Faculty of Medicine, Imperial College London, Hammersmith Hospital, London, United Kingdom; The Jackson Laboratory, United States of America

## Abstract

Differentiated cells can be reprogrammed through the formation of heterokaryons and hybrid cells when fused with embryonic stem (ES) cells. Here, we provide evidence that conversion of human B-lymphocytes towards a multipotent state is initiated much more rapidly than previously thought, occurring in transient heterokaryons before nuclear fusion and cell division. Interestingly, reprogramming of human lymphocytes by mouse ES cells elicits the expression of a human ES-specific gene profile, in which markers of human ES cells are expressed (hSSEA4, hFGF receptors and ligands), but markers that are specific to mouse ES cells are not (e.g., Bmp4 and LIF receptor). Using genetically engineered mouse ES cells, we demonstrate that successful reprogramming of human lymphocytes is independent of Sox2, a factor thought to be required for induced pluripotent stem (iPS) cells. In contrast, there is a distinct requirement for Oct4 in the establishment but not the maintenance of the reprogrammed state. Experimental heterokaryons, therefore, offer a powerful approach to trace the contribution of individual factors to the reprogramming of human somatic cells towards a multipotent state.

## Author Summary

One of the most pressing objectives of medical research today is the development of approaches to restore the function of tissues damaged by accident or disease. An important goal for this work is the isolation of stem cell populations to replace missing or nonfunctioning cells. Because problems of immune rejection are likely to occur unless the recipient and donor stem cells are very closely matched, a desirable strategy is to convert differentiated cells (such as white blood cells) from patients into immature tailored stem cell populations. Here, we have experimentally fused human white blood cells and mouse embryonic stem cells and shown that this reprograms them to become stem-like. This kind of “differentiation reversal” is shown to be rapid and stable. It requires the stem cell–specific factor Oct4, but does not require Sox2. This approach allows the identification of factors that are required to reprogram human blood cells with the long-term perspective to eventually generate patient-specific stem cells.

## Introduction

Reprogramming somatic cells to become ES-like is an important goal in cell replacement therapy since it affords the opportunity to generate and use patient-specific ES derived cells as grafts. Epigenetic reprogramming can be achieved in different ways including nuclear transfer [1]–[4] or the forced expression of one or more transcription factors [5],[6]. Retroviral-mediated expression of four transcriptional regulators, Oct4, Sox2, c-Myc and Klf4, was shown to drive mouse fibroblasts into an ES-like (iPS) state, albeit at low frequency [7],[8]. Reprogramming of human fibroblasts has also recently been achieved in a parallel approach using Oct4, Sox2 and either Nanog plus Lin28 [9] or Klf4 plus c-Myc [10].These pioneering studies have illustrated the importance of several factors for iPS, but also suggested that additional ones may be needed for efficient conversion to pluripotency. Reprogramming can also be achieved by cellular fusion, a process that occurs spontaneously *in vitro* [11], *in vivo* [12] and experimentally using specific agents [13]. For example, fusion of differentiated cells with pluripotent ES cells, embryonic carcinoma (EC) or embryonic germ (EG) cells, induces the expression of pluripotency-associated markers in the hybrid cells [14]–[18] and chromatin remodelling at specific sites in the somatic cell genome [14], [15], [18]–[21]. While these data show that reprogramming occurs through the epigenetic resetting of gene expression programs in the differentiated cell, it has been unclear whether nuclear fusion and genome duplication are absolutely required for successful conversion [22]. Here we investigated the requirements for, and the stability of, dominant reprogramming of human B cells by fusion with mouse ES cells. We show that reprogramming is surprisingly rapid and occurs within heterokaryons in which lymphocyte and ES cell nuclei remain spatially discrete. Furthermore, our data show that while Oct4 is critical for successful reprogramming of human lymphocytes to an ES-like state, Sox2 is not required. Thus our data outline an alternative strategy for defining the factors that are required for inducing a pluripotent state in human somatic cells.

## Results

### Reprogramming of Gene Expression Is Initiated in ES Cell Heterokaryons Prior to Nuclear Fusion

**Figure 1:**
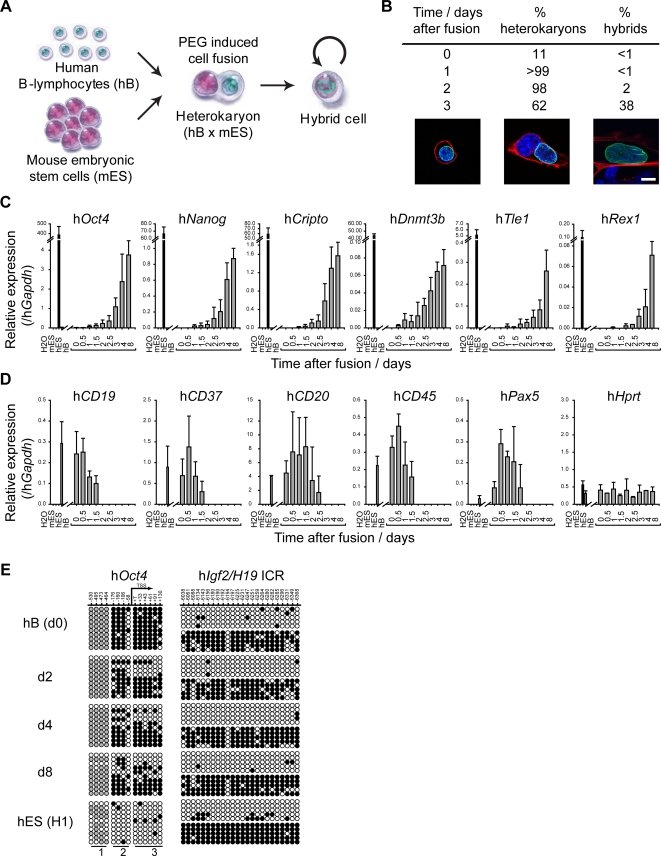
Pluripotent reprogramming of human B-lymphocytes by mouse ES cells is initiated in heterokaryons prior to nuclear fusion and cell division. (A) Shows the experimental strategy used to generate interspecies heterokaryons (hB x mES). Human B-lymphocytes (hB) and mouse embryonic stem cells (mES) were respectively labelled with the cell membrane dyes DiI and DiD and fused in the presence of polyethylene glycol (PEG). Fused cells were FACS sorted and cultured under conditions that promote mES self-renewal. (B) Immunofluorescence analysis of the kinetics of heterokaryon (cells in which parental nuclei share the same cytoplasm but remain discrete) and hybrid formation (where both parental genomes occupy the same nucleus) following PEG induced fusion. In the lower panels hB-derived nuclei were distinguished by mouse nuclei on basis of DAPI (blue) and human Lamin A/C staining (green), and actin staining (red) delineates individual cells. Confocal sections showing a hB cell prior to fusion (left, day 0), a heterokaryon [one mouse (with DAPI intense foci) and one human nucleus (hLamin A/C positive)] (middle, day 2) and a hybrid cell (right, day 3) are shown. Scale bar, 10 µm. *n* = 100. (C) The expression of hES-specific genes (h*Oct4*, h*Nanog*, h*Cripto*, h*Dnmt3b*, h*Tle1,* h*Rex1*) was assessed by quantitative RT-PCR analysis 0 to 8 days after cell fusion. Positive (hES-NCL1, black bars) and negative (hB) controls for this analysis were included. (D) Activation of embryonic genes is accompanied by silencing of lymphocyte-specific genes (h*CD19*, h*CD37*, h*CD20*, h*CD45* and h*Pax5*), while a constitutively expressed gene h*Hprt* remained detectable at similar levels at all time points. Data were normalised to h*Gapdh* expression. Error bars indicate the s.d. of 3 independent experiments. (E) Bisulfite genomic sequencing analysis of DNA methylation at the human *Oct4* promoter 0, 2, 4 and 8 days after cell fusion demonstrated the rapid de-methylation of *Oct4* induced by fusion with mES cells. Human ES cells (hES, cell line H1; lower panel) are shown as controls. The methylation pattern of *Igf2/H19* imprinting control region (ICR) remained unaltered throughout the experiment. The position of CpG sites relative to the transcriptional start site (TSS) is indicated. Open circles represent unmethylated cytosines, black closed circles represent methylated cytosines and grey closed circles represent constitutively methylated cytosines. Regions 1, 2 and 3 indicate CpG sites that are part of the same PCR product.

Human B cells were fused with mouse ES cells using polyethylene glycol (PEG). The nuclear events in fused cells were monitored by fluorescence microscopy and quantitative RT-PCR to analyse gene expression (Figure 1). To facilitate the identification of fused cells, E14tg2a mouse ES cells were pre-labelled with DiD and human B cells with DiI and dual-stained cells were purified by FACS (typically 10–15% of cells, Figure S1A). Human (B cell-derived) and mouse (ES cell-derived) nuclei were distinguished on the basis of DAPI and human-specific Lamin A/C labelling, and the proportion of cells containing two discrete (heterokaryons) or conjoined nuclei (hybrids) was assessed over time (Figure 1B). Up to 2 days following cell fusion 98–99% of dual labelled cells were identified as heterokaryons in which a single human and a single mouse nucleus were evident (illustrated in Figure 1B, central image). The kinetics of nuclear fusion were also confirmed by fluorescence *in situ* hybridization (FISH) analysis in which probes specific for mouse chromosomes (γ-satellite, red) or human chromosomes (α-satellite, green) were used to detect interspecies chromosome mixing, indicative of hybrid formation (Figure S1B and Text S1).

The expression of pluripotency-associated genes and lymphocyte-associated genes by human B cell-derived nuclei was assessed by qRT-PCR, using primers that selectively amplify the human transcripts. Expression of human *Oct4*, *Nanog*, *Cripto, Dnmt3b* and *Tle1* was detected in cells as early as 1 day after fusion and human *Rex1* after 2 days (Figure 1C and Figure S1C). Although the levels were low in heterokaryons (<1% of that detected in human ES cells, cell line NCL1), these increased over time and were undetectable in non-fused (or self-fused, not shown) human B cells or control mouse ES cells. Expression of h*Tert* was detected from day 4 onwards (Figure S1D), while h*Hprt* expression was equivalent at all stages, as anticipated (Figure 1D). Mouse lymphocyte-specific gene transcripts (m*CD19*, m*CD37* and m*CD45*) were not detected throughout the analysis (not shown), confirming the dominance of ES cells in conversion [15],[18]. Increased expression of human pluripotency-associated genes over this 8-day period was mirrored by a reduction in expression of several human lymphocyte-associated genes within the second (h*CD45*, h*CD37* and h*CD19*) or third day (h*CD20* and h*Pax5*) of heterokaryon formation (Figure 1D). Collectively these data show that upon dominant reprogramming, activation and silencing of tissue-specific gene programs begins ahead of, and therefore does not require, nuclear fusion and cell division. In addition, since these results examine gene expression at the population level, it is possible that gene expression varied between individual heterokaryons and hybrid cells.

As the reprogramming of somatic cells has been previously shown to result in altered DNA methylation at specific loci [15],[18],[21],[23], we examined changes in the methylation status of the human *Oct4* gene promoter [24] and as a control, the *Igf2*/*H19* imprinting control region (ICR) [25]. As illustrated in Figure 1E, human B cells prior to fusion showed high levels of DNA methylation throughout the h*Oct4* promoter and across a single *Igf2*/*H19* allele. Following cell fusion, DNA methylation of h*Oct4* in reprogrammed B cells declined, consistent with a trend towards a hypomethylated state as seen in the human ES cell line H1. Demethylation of the h*Oct4* promoter was detected prior to nuclear fusion and cell division, a result that is consistent with active chromatin remodelling of the locus prior to expression. No changes in DNA methylation at *Igf2*/*H19* ICR were detected over this period, consistent with its imprinted status [25].

### Induction of a Human ES-Specific Gene Expression Profile

**Figure 2:**
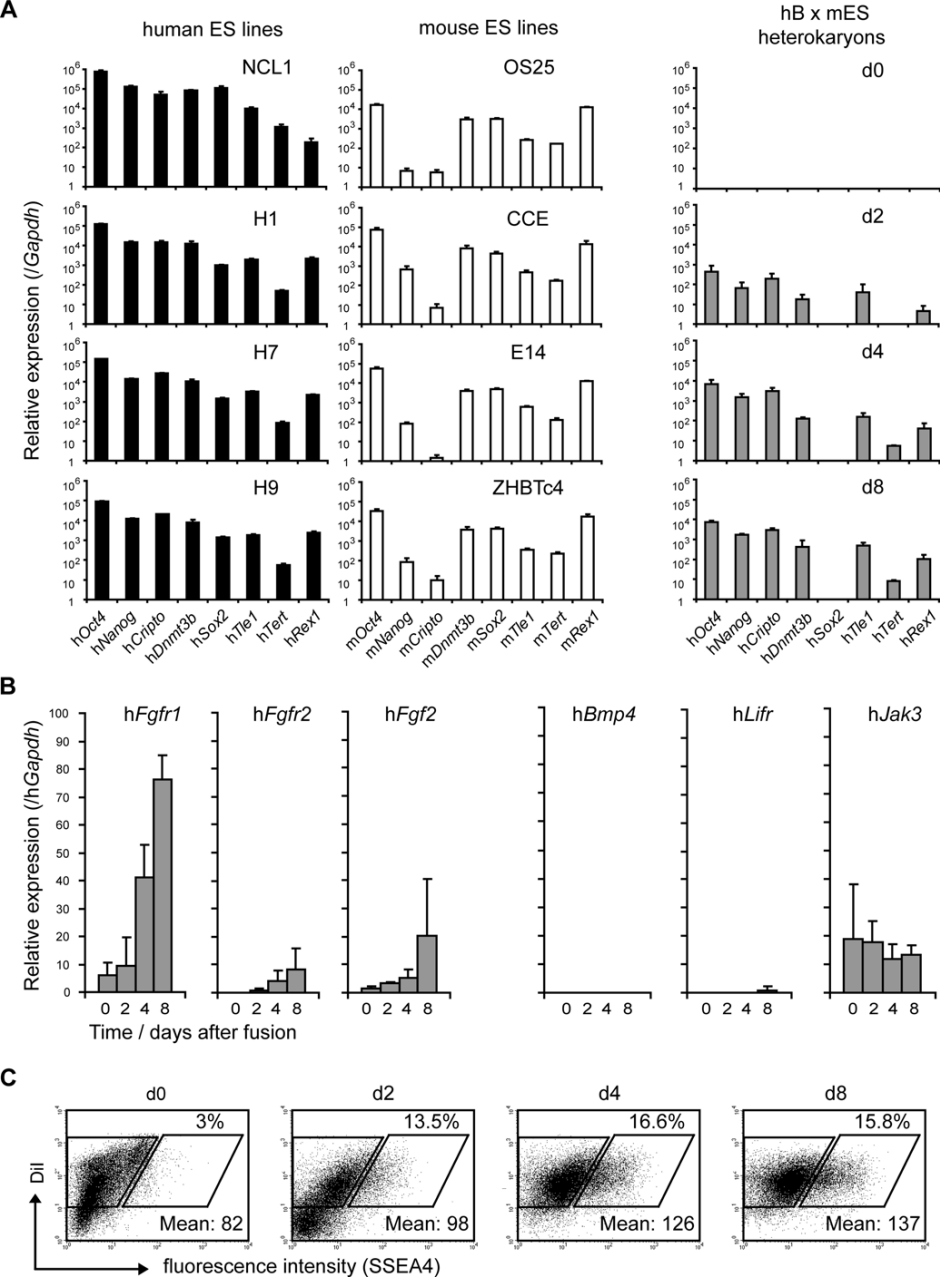
Gene expression in reprogrammed lymphocytes resembles human rather than mouse ES cell lines. (A) Quantitative RT-PCR analysis of the relative levels of gene expression in several human (NCL1, H1, H7 and H9), mouse (OS25, CCE, E14 and ZHBTc4) ES cell lines and in reprogrammed lymphocytes (hB x mES) at 0, 2, 4 and 8 days after cell fusion. Human ES lines (left panel) and hB x mES (right panel) gene expression data were normalised to h*Gapdh*. Mouse ES lines (middle panel) gene expression data were normalised to m*Gapdh*. Error bars indicate the s.d. of 3–4 independent experiments. (B) After cell fusion, genes involved in the maintenance of undifferentiated human ES cells (h*Fgfr1,* h*Fgfr2* and h*Fgf2*) were activated while genes selectively expressed by mouse ES cells (h*Bmp4*, h*Lifr* and h*Jak3*) were not induced. Data were normalised to h*Gapdh* expression. Error bars indicate the s.d. of 3 independent experiments. (C) Heterokaryons resulting from human B cell and mouse ES cell fusions (hB x mES) were stained for SSEA4 at 0, 2, 4 and 8 days and expression was analysed by flow cytometry. The results showed that 13.5% (day 2), 16.6% (day 4) and 15.8% (day 8) of total heterokaryons expressed SSEA4, as delineated by the rhomboid gates. Mean intensity fluorescence of positive cells is indicated.

A comparison of the relative abundance of gene-specific transcripts in reprogrammed human B cells (Figure 2A, right-hand column), showed a strong similarity with the gene expression profiles of several human ES cell lines (NCL1 [26], HI, H7, H9 [27]; Figure 2A, left-hand column). For example, while *Oct4* was abundantly expressed in all human and mouse ES cell lines, *Nanog* and *Cripto* expression was consistently much lower than *Oct4* (100–1000 fold) for each of the mouse ES cell lines analysed (OS25, CCE, E14, ZHBTc4; Figure 2A, middle panel). In human ES cell lines however, *Oct4*, *Nanog* and *Cripto* transcripts were similarly abundant, consistent with that seen in reprogrammed human B cells. Expression of some pluripotency-associated genes, for example *Sox2*, was variable and often required extended periods of time (>8 days) for detection (not shown). This could reflect the fact that genes such as *Sox2* are subject to multiple layers of repressive epigenetic modifications in B cells including DNA and histone methylation [28],[29] and late replication [30], or that they require a higher threshold of activators for overt expression.

Similarities between gene expression profiles of human ES cell lines and hB x mES fused cells prompted us to examine additional markers that are expressed solely by either human or mouse ES cells [31]–[34]. These included fibroblast growth factor receptors (Fgfr1 and Fgfr2) and Fgf2 (expressed by human ES cells), Bmp4 and leukaemia inhibitory factor (Lif) receptor (expressed by mouse ES cells) and SSEA4, a surface glycoprotein selectively expressed by human ES cells [27] (Figure S2B). This analysis revealed that reprogrammed cells expressed increasing amounts h*Fgfr1*, h*Fgfr2* and h*Fgf2* but did not express h*Bmp4* or h*Lifr* or upregulate the downstream kinase h*Jak3* (Figure 2B). Thus, these data show that while dominant conversion is driven by mouse ES cells (that express *Bmp4* and *Lifr* prior to fusion, Figure S2A), reprogrammed heterokaryons and hybrid cells show a remarkably different expression profile resembling human, rather than mouse ES cell lines. Consistent with this, fusion of mouse ES cells and human B cells resulted in SSEA4 expression by 13–16% of the cells (days 2–8 as shown in Figure 2C). Isolation of SSEA4-positive cells confirmed that this subset contained successfully reprogrammed cells that express h*Oct4*, h*Nanog* and h*Cripto* (Figure S2C), while SSEA4-negative cells were not reprogrammed. The observation that only a proportion of heterokaryons are successfully reprogrammed, as judged by h*Oct4* DNA demethylation and SSEA4 expression, might partly explain why the levels of transcripts encoding pluripotency factors are lower in reprogrammed cultures than established hES cell lines.

**Figure 3:**
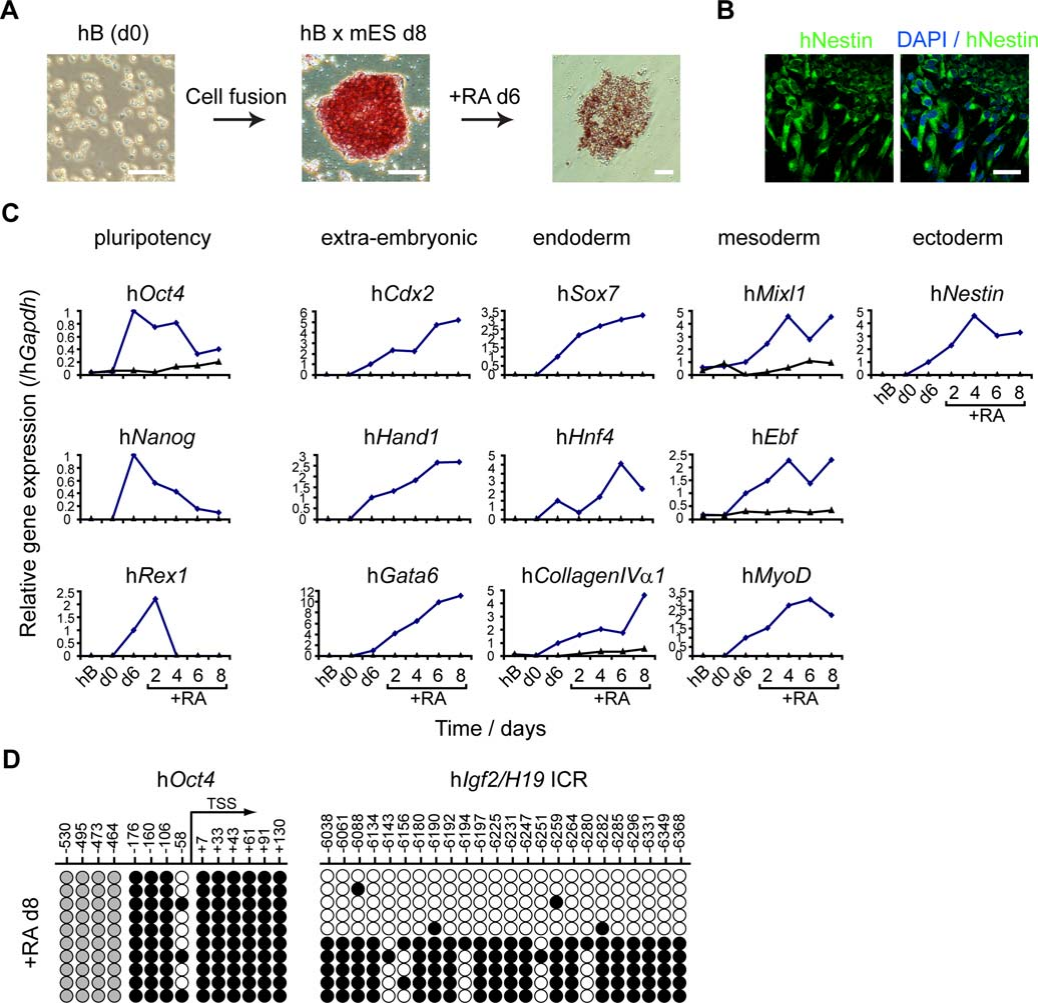
Multi-lineage potential is reset in reprogrammed human lymphocytes. (A) Hybrid colonies resulting from fusion of human B cells (hB) and mouse ES cells (hB x mES) showed alkaline phosphatase activity (pink), that was reduced upon retinoic acid (RA) treatment. (B) RA treatment of hybrid colonies (day 6) generated cells that expressed Nestin (green) detected by immunostaining using an antibody specific for human (and not mouse) Nestin protein. DAPI counterstaining (blue) is shown. Scale bars, 50 µm. (C) Quantitative RT-PCR analysis of gene expression upon RA treatment of hybrid cells (blue line) showed that levels of pluripotency genes (h*Oct4*, h*Nanog* and h*Rex1*) declined while differentiation-associated genes were upregulated [extra-embryonic (h*Cdx2*, h*Hand1* and h*Gata6*), endoderm (h*Sox7*, h*Hnf4* and h*CollagenIVαI*), mesoderm (h*Mixl1*, h*Ebf* and h*MyoD*) and ectoderm (h*Nestin*)]. Unfused hB cells were included as controls (black line). Data were normalised to h*Gapdh* expression. (D) Bisulfite genomic sequencing analysis of DNA methylation at the human *Oct4* promoter 8 days after RA treatment showed the re-methylation of the *Oct4* promoter while the *Igf2/H19* imprinted control region (ICR) remains unaltered. The position of CpG sites relative to the transcriptional start site (TSS) is indicated. Open circles represent unmethylated cytosines, black closed circles represent methylated cytosines and grey closed circles represent constitutively methylated cytosines.

To ask whether the reprogramming of human B cells by mouse ES cells resets multi-lineage potential, hB x mES cultures were treated with retinoic acid (RA) 6–8 days after cell fusion in order to induce differentiation (Figure 3). Prior to RA treatment most cells in hB x mES colonies showed alkaline phosphatase (AP) activity (Figure 3A), and expressed human AP transcripts (not shown). Hybrid colonies also expressed several pluripotency-associated markers, including hNanog protein (detected using a human Nanog-specific antibody) and the human embryonic-specific antigens SSEA4, TRA-1-60 and TRA-1-81 [27] (Figure S3). Following treatment with RA, AP activity and expression of h*Oct4*, h*Nanog* and h*Rex1* was reduced (Figure 3C), while morphological heterogeneity within colonies increased. RA treatment induced the expression of genes associated with extra-embryonic (h*Cdx2*, h*Hand1* and h*Gata6*), endoderm (h*Sox7*, h*Hnf4* and h*CollagenIVαI*), mesoderm (h*Mixl1*, h*Ebf* and h*MyoD*) and ectoderm (h*Nestin,* Figure 3B) differentiation in hB x mES, but not in control hB cells (Figure 3C, blue and black lines respectively). Differentiation also resulted in increased DNA methylation of the h*Oct4* promoter (Figure 3D) to levels similar with that seen in differentiated human cells (Figure 1E). Taken together, these results show that reprogramming of human B cells by mouse ES cells resets gene expression and multi-lineage potential.

### Interspecies Reprogramming of Human B Cells Requires m*Oct4* but Not m*Sox2*

**Figure 4:**
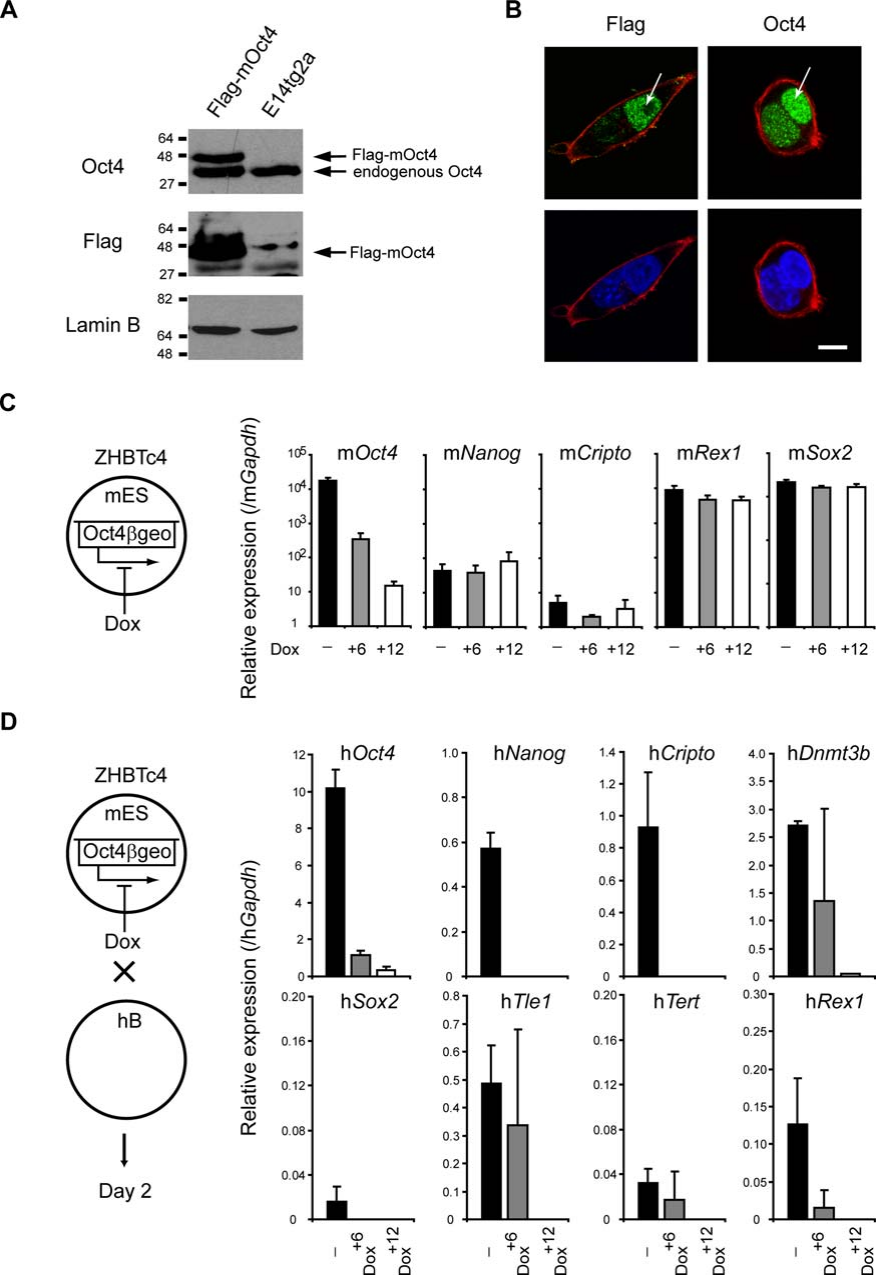
Oct4 is required for successful reprogramming. (A) Mouse ES cells expressing a tagged Oct4 protein (Flag-mOct4) were generated by insertion of Flag-tagged mouse *Oct4* cDNA in E14tg2a ES cells (parental cell line). Western blotting with anti-Oct4 and anti-Flag antibodies confirmed the presence of Flag-tagged Oct4 protein by transduced cells. Equivalent protein loading is shown with Lamin B detection. (B) Immunofluorescence analysis of cultured heterokaryons 6 hours after cell fusion showed the presence of ES cell-derived Oct4 (Flag-Oct4, green) in a human nucleus (arrowed). Human nuclei were distinguished from mouse nuclei on basis of diffuse versus punctuate DAPI staining (blue), respectively. Actin labelling (red) delineates the cell membrane. Images are confocal sections of heterokaryons containing a single mouse (with DAPI intense foci) and a single human nucleus. Scale bar, 10 µm. (C) In ZHBTc4 ES cells endogenous *Oct4* was replaced by an inducible transgene (Oct4βgeo) which can be downregulated by addition of doxycycline (Dox) [36]. Quantitative RT-PCR analysis showed that 6 hours (+6) and 12 hours (+12) after Dox treatment, m*Oct4* was progressively downregulated, while expression of other pluripotency-associated genes (m*Nanog*, m*Cripto*, m*Rex1* and m*Sox2*) was largely unaffected. (D) ES cells expressing normal levels of Oct4 (-), partially reduced (Dox^+6^) or lacking Oct4 expression (Dox^+12^) were fused to hB-lymphocytes. Successful reprogramming was assessed by quantifying the abundance of human ES-associated transcripts two days after fusion by qRT-PCR. Activation of pluripotency genes in hB-lymphocytes was reduced or impaired when Oct4 was ablated. Data were normalised to *Gapdh* expression. Error bars indicate the s.d. of 2–3 independent experiments.

Oct4 is part of the core regulatory circuitry in ES cells [35] and it is essential for pluripotency and self-renewal [36]. To assess the potential role of mouse-derived Oct4 as a dominant ‘*trans*’ acting factor within inter-species heterokaryons we generated ES cells expressing Flag-tagged mouse Oct4 protein (Figure 4A) and fused these with human B lymphocytes (Figure 4B). Flag-tagged Oct4 (derived from mouse ES cells) was seen to accumulate within human nuclei 3 to 6 hours after cell fusion (Figure 4B; complete kinetic experiment shown in Figure S4A). In addition, Oct4 protein was present in heterokaryon nuclei (at 3 hours) before transcription of h*Oct4* was initiated (at 24 hours). Thus, translocation of the ES-derived Oct4 into human lymphocyte nuclei precedes reprogramming.

**Figure 5:**
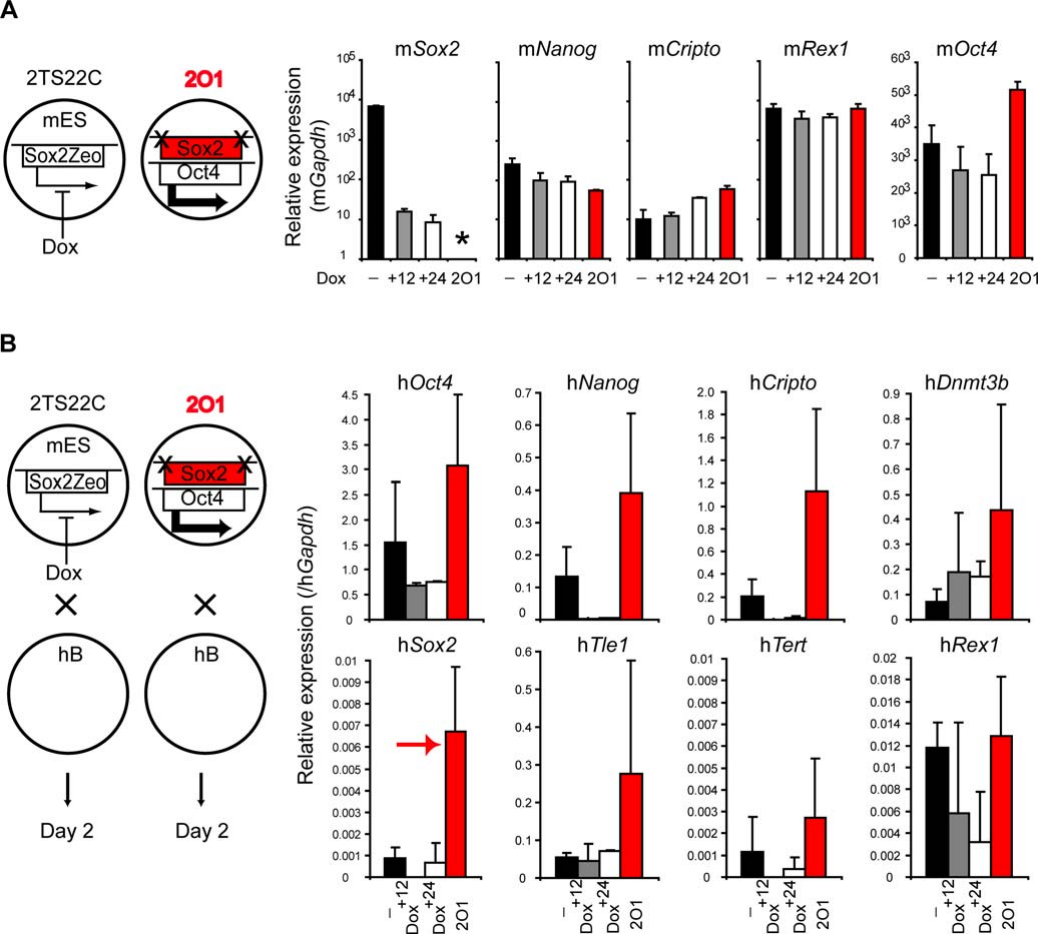
Sox2 is dispensable for reprogramming. (A) In 2TS22C ES cells endogenous *Sox2* is replaced by an inducible transgene (Sox2Zeo) which can be downregulated by addition of doxycycline (Dox) [37]. Quantitative RT-PCR analysis showed that 12 hours (+12) and 24 hours (+24) after Dox treatment, m*Sox2* was downregulated while expression of other pluripotency-associated genes (m*Nanog*, m*Cripto*, m*Rex1* and m*Oct4*) continued to be expressed. 2O1 ES cells are Sox2-deficient mES cells (asterisk) in which m*Oct4* expression is up-regulated (red bars). (B) ES cells expressing Sox2 (-), Sox2 depleted cells (Dox^+12^, Dox^+24^) and 2O1 cells were fused to hB-lymphocytes. Successful reprogramming was assessed by quantifying the abundance of human ES-associated transcripts two days after fusion by qRT-PCR. Activation of pluripotency genes in hB-lymphocytes occurs in the absence of mSox2. An elevated induction of h*Sox2* using 2O1 cells as a fusion partner is highlighted by an arrow (red). All data were normalised to *Gapdh* expression and error bars indicate the s.d. of 2–3 independent experiments.

Conversion of human fibroblasts to ES-like cells has been shown to require the activation of at least four factors including Oct4, Sox2 and either Nanog plus Lin28 [9] or Klf4 plus c-Myc [10]. Recently it was shown that mouse ES cells lacking Sox2, a factor thought to be vital for preventing extra-embryonic differentiation, can remain pluripotent provided with elevated Oct4 levels [37]. To investigate the relative importance of Oct4 and Sox2 in reprogramming, mouse ES cells that are inducible null (Tet-off) for m*Oct4* (ZHBTc4 [36]) or for m*Sox2* (2TS22C [37]) were used as fusion partners with human B cells. These inducible null ES cell lines were constructed and characterised previously [36],[37] and display a rapid (within 24 hours) and complete elimination of Oct4 or Sox2 gene/protein expression upon doxycycline (+Dox) treatment. In our hands, pre-treatment of ZHBTc4 cells with Dox for 6 and 12 hours, resulted in a progressive decrease in m*Oct4* gene expression (Figure 4C), without significantly affecting the expression of other pluripotency-associated genes in these cells or the efficiency which they fuse with human B cells (Figure S4B). Successful reprogramming, as judged by induction of several human genes (*Oct4*, *Nanog*, *Cripto*, *Dnmt3b*, *Sox2*, *Tle1*, *Tert* and *Rex1*) was however reduced (+6 hours) or eliminated (+12 hours) by pre-treatment of ZHBTc4 cells with Dox (Figure 4D, a complete kinetic analysis is provided in Figure S4C). Likewise, knocking down m*Oct4* using short interference RNA (siRNA) in E14tg2a mES cells (Figure S5A and Text S1) also abolished reprogramming activity (Figure S5B). These results confirm that m*Oct4* expression is critically important for initiating successful reprogramming, in keeping with previous reports [7]–[10],[38]. The extinction of human lymphocyte-specific genes was however not impaired by Oct4 removal (Figure S4C), a result that may support previous findings that the activation and silencing of gene expression programs in heterokaryons are mechanistically distinct processes [13]. Eliminating m*Sox2* expression in the mouse ES cell (Figure 5A, 2TS22C) had, in contrast, a relatively mild effect on reprogramming efficiency (Figure 5B, compare values at 0, 12 and 24 hours of Dox treatment). Furthermore, reprogramming was fully restored in fusions using 2O1 cells, a Sox2-deficient mES cell line in which m*Oct4* expression is up-regulated [37] (Figure 5A,B values shown in red and complete kinetics shown in Figure S6). These data show that Oct4, but not Sox2, is critical for the dominant reprogramming activity of mouse ES cells. Interestingly, using 2O1 cells we observed the enhanced induction of h*Sox2* (Figure 5B, red arrow), a result that suggests that mouse-derived Oct4 levels may be important for initiating h*Sox2* expression in somatic nuclei.

### ES-Derived m*Oct4* Is Dispensable for Maintaining the Reprogrammed Status of Somatic Cells

**Figure 6:**
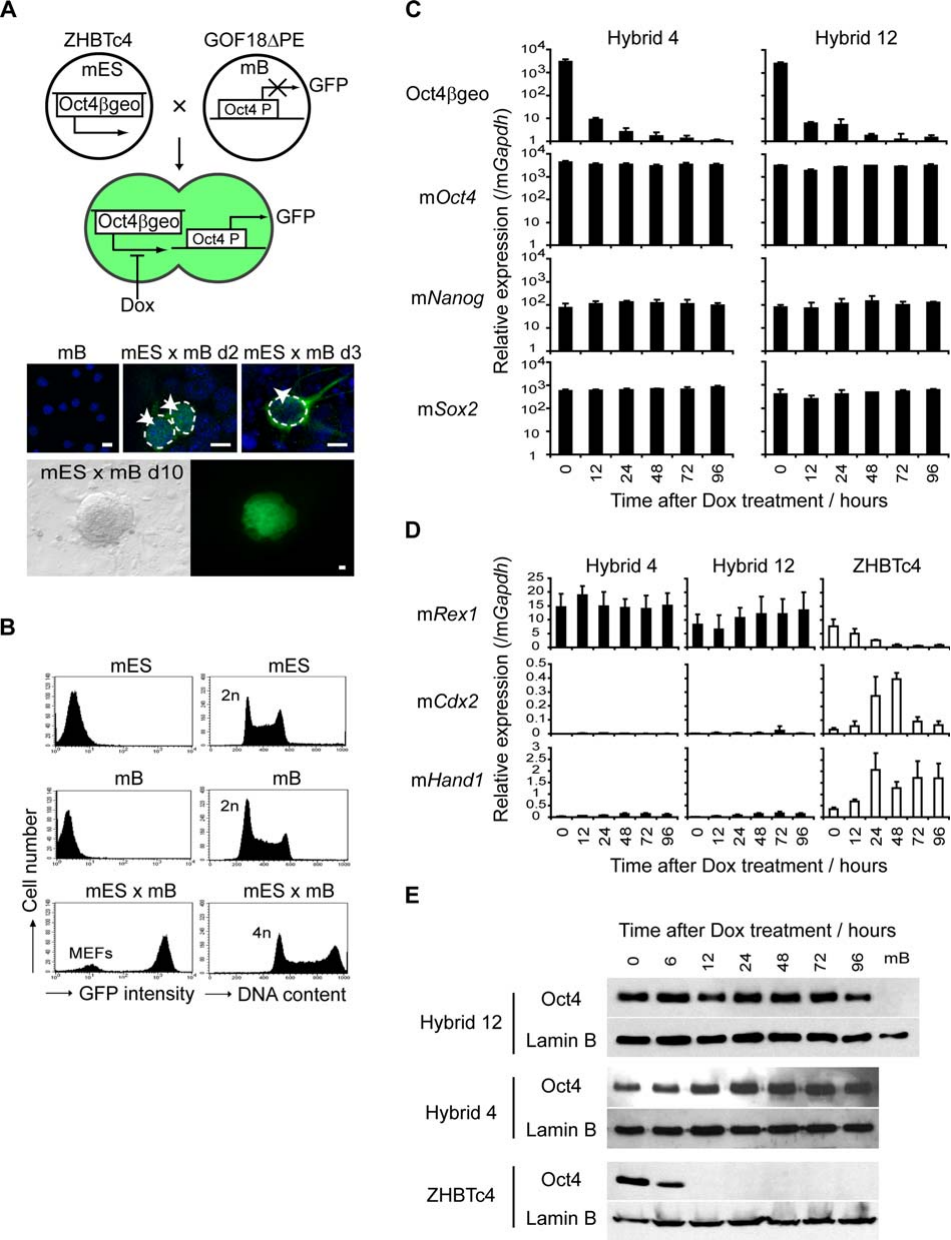
Reprogramming is self-sustaining and can be maintained in the absence of ES-derived Oct4. (A) To address whether reprogramming is stable or subject to reversion, we ablated *Oct4* expression after hybrid formation. ZHBTc4 ES cells [mES with endogenous *Oct4* replaced by an inducible transgene (Oct4βgeo) which can be downregulated by addition of doxycycline (Dox)] were fused with mouse B-lymphocytes (mB) carrying a GFP transgene under the control of Oct4 promoter (GOF18ΔPE). Reprogramming of mB results in the re-activation of GFP in hybrid colonies (d10, lower panels). Kinetic analysis of single cells (upper panels) showed that transgene re-activation occurs in heterokaryons (day 2, 2 arrows), and hybrid cells (day3, arrowhead). mB cells are shown as negative controls. Nuclei were visualised with DAPI staining (blue). Scale bars, 10 µm. (B) Hybrid clones (mES x mB, 4n) that re-expressed GFP were isolated and analysed by FACS. mES, mB and mES x mB hybrid cells unstained (left panel) or stained with propidium iodide (right panel) to assess GFP expression and DNA content, respectively. (C) Hybrid clones (4 and 12) were treated with Dox to ablate ES-derived *Oct4*, and quantitative RT-PCR confirmed downregulation of Oct4βgeo transcript (upper panels). Removal of mES-derived Oct4βgeo in hybrid clones did not affect gene expression of pluripotency-associated transcripts (lower panels; m*Oct4*, m*Nanog* and *mSox2*) after 96 hours of Dox treatment. (D) No differentiation was observed after *Oct4* removal in hybrid cells. m*Rex1* expression was retained and the extra-embryonic markers m*Hand1* and m*Cdx2* were not induced. In ZHBTc4 ES cells (open bars) upon Dox treatment, m*Hand1* and m*Cdx2* were induced [36] and are shown for comparison. Data were normalised to m*Gapdh* expression. Error bars indicate the s.d. of 3 independent experiments. (E) Western blotting with anti-Oct4 antibody confirmed that Oct4 protein is rapidly removed after Dox treatment of ZHBTc4 ES cells (lower panel) but remains detectable at all times in hybrid cells (upper panels). Equivalent protein loading is shown with Lamin B detection.

To assess whether gene expression by the reprogrammed cell is stable (self sustaining) or requires the continuous supply of factors provided by the mouse ES cell, we generated hybrid cells between lymphocytes and ES cells in which Oct4 expression could be conditionally withdrawn (ZHBTc4, experimental outline depicted in Figure 6A). In these experiments fusions were performed between mouse lymphocytes carrying a silent, Oct4-driven GFP transgene (GOF18ΔPE) and mouse ZHBTc4 ES cells, to allow successfully reprogrammed hybrid cells to be identified on the basis of GFP re-expression by day 10 (Figure 6A). Hybrid clones contained a rearranged *IgH* locus, consistent with their derivation from mouse B cells (Figure S7A and Text S1), displayed twice the DNA content of diploid cells (4n, Figure 6B) and were karyotypically stable over the study period (not shown). As anticipated, hybrid cells expressed ZHBTc4-derived Oct4βgeo transcripts and several pluripotency-associated genes, but did not express B cell markers such as *CD19*, *Pax5* and *Ly108* (Figure S7B). Two hybrid clones were selected for study (hybrid 4 and 12) and were treated with Dox to selectively ablate expression of ZHBTc4-derived Oct4βgeo (Figure 6C; Figure S7C shows the strategy used to selectively detect Oct4βgeo transgene expression). Withdrawal of ZHBTc4-derived Oct4 did not alter the expression of m*Nanog* and m*Sox2* in reprogrammed cells (Figure 6C), and did not precipitate differentiation towards trophectoderm or the up-regulation of m*Cdx2* and m*Hand1* expression [36] (Figure 6D and Figure S7D); events that are induced by the removal of Oct4 from the parental ZHBTc4 line (Figure 6D right hand panel and Figure 6E). Thus, our data show that reprogramming of lymphocytes by mouse ES cells induces an epigenetically stable (and heritable) resetting of gene expression in the lymphocyte nucleus.

## Discussion

In this study we show that reprogramming human lymphocytes can be achieved using mouse ES cells as a cell fusion partner, a process that induces the re-expression of endogenous human genes normally associated with human blastocyst development and human ES cell lines. Successful interspecies reprogramming is initiated in heterokaryons prior to chromosome intermixing, and generates cells that express human FGF signalling pathway components and human ES-specific surface molecules such as SSEA4, TRA-1-60 and TRA1-81. We show that this reprogramming is critically dependent upon Oct4, since Oct4 deletion abolishes the reprogramming capacity of mES cells. Conversion of human B cells into ES-like cells results in the re-modelling of the somatic genome with loss of DNA methylation at the h*Oct4* locus. Importantly, once reprogramming is initiated by factors produced by the dominant (ES) nucleus, we show that withdrawal of mOct4 does not compromise the phenotype of hybrid cells. This result implies that the reprogrammed state, once initiated, is both self-sustaining and heritable.

One surprising aspect of the reprogramming data shown here is the rapidity of gene conversion and DNA demethylation that occurs within heterokaryons. As successful reprogramming is only achieved in a proportion of heterokaryons (<13%), it is likely that partial DNA replication (or repair) is required for lymphocyte conversion. Previous studies have shown that reprogramming in experimental heterokaryons using adult cells from different lineages [13],[39], can be initiated before genome duplication and cell division. Here we show that conversion of unipotent lymphocytes towards multipotency is achieved in transient heterokaryons prior to cell division. Re-activation of human *Oct4* and *Nanog* by human nuclei, has been shown to occur rapidly upon DNA de-methylation and Tpt1 activation induced by *Xenopus* oocytes [21],[40]. The rapid re-activation of endogenous pluripotency-associated genes seen in inter-species heterokaryons is consistent with transgene re-activation studies that have reported *Oct4gfp* expression by MEFs [41] or NSCs [22] fused with mouse ES or EC cells. Collectively these results may have an impact for generating human ES-like cells. Proof that mouse ES cells can dominantly reset the multi-lineage potential in human somatic cells, together with evidence that this process begins prior to nuclear fusion, suggests that improved methods for removing mouse chromosomes from heterokaryons [42] may be applicable for generating human stem cell lines. Alternatively, using conditionally targeted mouse ES cells to dissect the roles of individual proteins thought to be critical for multipotent reprogramming, may provide a rationale for using distinct protein cocktails to directly re-set lineage potential.

In the experiments presented here we have shown that reprogrammed human cells express a profile of transcripts, signalling molecules and surface antigens that are similar to those seen in human ES cells, and different from mouse ES cells. This suggests that an early human embryonic “program” of gene expression is initiated in human nuclei by *trans*-acting (mouse) factors. Differences between the expression profiles of mouse ES cells and human reprogrammed nuclei probably reflect discrepancies in *cis*-acting regions between the mouse and human genomes. In agreement with this idea, a study in which the entire h*Tert* gene was introduced into mice, showed expression of the transgene was similar to endogenous h*Tert* in humans, rather than mouse endogenous m*Tert* [43]. It is interesting to speculate that some of the well-publicised differences between human and mouse ES cells may indeed reflect intrinsic species dissimilarities, rather than temporal differences in stem cells isolation [44],[45]. We show that after fusion of human lymphocytes to mouse ES cells (that are Lif and Bmp dependent), human ES-like cells are generated that express FGF signalling components (and are not dependent of Lif/Bmp). Thus, our data suggest that differences between human and mouse ES cells may reflect distinct signalling and transcriptional networks, rather than necessarily when or where they were isolated during embryogenesis.

We show here that Sox2, in contrast to Oct4, is not required to convert human lymphocytes into a multi-potent state. This observation contrasts with results obtained previously using iPS strategies to reprogram mouse and human fibroblasts [7]–[10],[38], mouse hepatocytes and stomach cells [46] and mouse B-lymphocytes [47]. Whether this is because of differences relating to the overexpression of transcription factor cocktails used in iPS, or that reprogramming occurs over an extended time period (pluripotency-associated genes such as *Oct4*, *Nanog* and *Sox2* are reactivated after 2 weeks of transduction [48],[49]), is not known. However, as Sox2 was recently shown to be dispensable for the activation of Oct–Sox enhancers in mouse ES cells [37], it is also possible that additional Sox family members such as Sox4, Sox11 and Sox15, may have redundant functions with Sox2 in reprogramming. Interestingly, by enhancing Oct4 levels in Sox2-deficient ES cells (ES-2O1) we show elevated expression of h*Sox2* by reprogrammed human B cells. Recent genome-wide studies have shown that *Sox2* is a direct target of Oct4 in both human [35] and mouse [50] ES cells, a fact that could explain why h*Sox2* is efficiently reprogrammed using ES cells that overexpress m*Oct4*. In our hands, overexpression of exogenous Oct4 in lymphocytes did not induce pluripotent conversion (Pereira & Terranova, unpublished results), a finding that argues that additional chromatin remodelling factors, perhaps including those known to interact with Oct4 [51],[52] or associated with the process of DNA demethylation, may be critical for successful reprogramming. Collectively, our data show that interspecies heterokaryons can provide an interesting and complimentary approach to iPS, allowing the factors that are required to directly induce pluripotency to be defined individually and in combination.

## Materials and Methods

### Cell Culture

EBV-transformed hB clones were maintained in RPMI supplemented with 10% foetal calf serum (FCS), 2 mM L-glutamine and antibiotics (10 µg/ml Penicillin and Streptomycin). The Abelson transformed Oct4-GFP B-cell line was derived from the Oct4-GFP transgenic mice (GOF18ΔPE) [53] bone marrow, cloned and grown in RPMI supplemented with 20% FCS, non-essential amino acids, L-glutamine, 50 µM 2-mercaptoethanol, antibiotics and IL-7 (5 ng/ml; R&D systems, Minneapolis, MN). Mouse ES cells were grown and maintained undifferentiated either on irradiated SNL feeder layers (E14Tg2a, *Hprt* ^−/−^ ES cells; CCE and E14) or directly on 0.1% gelatin-coated surfaces (OS25, ZHBTc4 and 2TS22C feeder-free ES cell lines). ES cells were grown in KO-DMEM medium plus 10% FCS, non-essential amino acids, L-glutamine, 2-mercaptoethanol, antibiotics and 1000 U/ml of leukaemia inhibitory factor (ESGRO-LIF). Feeder-free ES cell lines were cultured in GMEM-BHK21 medium plus 10% FCS, non-essential amino acids, sodium pyruvate, sodium bicarbonate, 2 mM L-glutamine, 2-mercaptoethanol, antibiotics and 1000 U/ml of LIF. Doxycycline (1 µg/ml, Sigma) or Retinoic acid (10^−6^ M, Sigma) were added to the media when indicated. The Flag-mOct4 cell lines were derived by the overexpression of Flag-tagged mouse Oct4 in E14tg2a ES cells. Briefly, mouse *Oct4* cDNA was cloned in the pDFLAG-cDNAIII vector (Invitrogen). The cDNA, including two flag sequences at the 5′ end, was excised and sub-cloned into a suitable vector for expression in ES cells (pCBA), with expression driven by the chicken β-actin promoter. The vector was then linearised and transfected by electroporation into mouse ES cells. G418 selection (400 µg/ml; Invitrogen) was applied 48 hrs after and resistant clones were manually picked and screened by Western blot. Human ES cell lines H1, H7 and H9 cells [27] were cultured in medium conditioned by mitotically inactivated MEFs supplemented with 8 ng/ml bFGF (Peprotech, London, UK) on matrigel-coated plates, as previously described [54]. Cells were routinely passaged at a 1∶3 dilution by treatment with 200 U/ml collagenase IV (Invitrogen, Carlsbad, CA) and mechanical dissociation.

### Experimental Heterokaryons

Heterokaryons were generated by fusing ES cells and B-lymphocytes using 50% polyethylene glycol, pH7.4 (PEG 1500; Roche Diagnostics, Mannheim, Germany). Briefly, ES cells and hB-lymphocytes were respectively labelled with Vibrant 1,1′-dioctadecyl-3, 3, 3′, 3′ tetramethylindodicarbocyanine (DiD) and 1,1′-dioctadecyl-3, 3, 3′, 3′-tetramethylindocarbocyanine perchlorate (DiI) cell labelling solutions (Molecular Probes, Eugene, OR). Cells were resuspended at 1×10^6^ cells/ml in DMEM and labelled with 5 µl/ml of dye at 37°C, 15 min. ES and hB were then mixed in an appropriate ratio (ES∶hB ratio 1∶1; ES∶Oct4-GFPB ratio 1∶5), and were washed twice in PBS. The supernatant was completely removed and 1 ml of PEG (37°C) was added to the pellet of cells over 60 sec and incubated at 37°C for 90 sec with constant stirring. Then, 4 ml of serum-free medium (DMEM) were carefully added over a period of 3 min, followed by 10 ml of DMEM and incubation at 37°C for 3 min. After centrifugation (1350 rpm, 5 min), the pellet was allowed to swell in complete medium for 3 min. Cell mixtures were then resuspended and cultured under conditions promoting the maintenance of undifferentiated mouse ES cells at 0.5×10^6^ cells/cm^2^. To eliminate unfused hB cells, Ouabain (10^−5^ M; Sigma) was added to the medium 4 hours after cell fusion. When OS25, ZHBTc4 and 2TS22C cell lines were used, proliferating ES cells were eliminated by the addition of 10^−5^ M Ara-C (Cytosine β-D arabino furanoside; Sigma) 4–6 hours after fusion and then removed after 16 hours. When E14tg2a ES cells or derivatives were used, HAT (20 µM hypoxanthine, 0.08 µM aminopterine and 3.2 µM thymidine; Sigma) was added to the medium 24 hours after fusion.

### Quantitative RT-PCR Analysis

RNA extraction was performed using RNA-BEE reagent (Tel-Test Inc., Friendswood, TX) and residual DNA was eliminated using the DNA-free kit (Ambion, Austin, TX). 3 µg of total RNA was then reverse transcribed using Superscript First-Strand Synthesis system (Qiagen) with oligo (dT)_12-18_ (Invitrogen). cDNAs of interest were then quantified using real-time qPCR amplification. Real-time PCR analysis was carried out on a Opticon DNA engine using Opticon Monitor software (MJ Research Inc., Waltham, MA), running the following program: 95°C for 15 min, then 40 cycles of 94°C for 15 sec, 60°C for 30 sec, 72°C for 30 sec, followed by plate-read. PCR reactions included 2× Sybr-Green PCR Mastermix (Qiagen), 300 nM primers and 2 µl of template in a 35 µl reaction volume. Each measurement was performed in triplicate and data normalised according to *Gapdh* expression. Primers were designed with Primer Express software (Applied Biosystems) and tested for the specific detection of human transcripts (and not mouse). Standard curves were calculated on serial dilutions of positive control cDNA. Primer sequences used for this analysis are indicated in Table S1.

### Bisulfite Genomic Sequencing

Bisulfite modification of DNA was carried out with the EZDNA methylation kit (Zymogenetics Inc., Orange, CA) according to manufacturer's recommendations. PCR primers that recognise bisulfite-converted human DNA only are listed in Table S1. Amplified products were cloned into pCR2 (Invitrogen) and ten clones were randomly picked and sequenced.

### Antibodies, Imaging, and FACS Analysis

For immunofluorescence and FACS analysis, the following antibodies and dilutions were used: mouse monoclonal anti-human Lamin A/C (VP-L550; Vector Laboratories Inc., Burlingame, CA) at 1∶100 dilution; rabbit polyclonal anti-GFP (A11122; Molecular Probes) at 1∶200 dilution; mouse monoclonal anti-human SSEA4 (MC-813-70; Developmental Hybridoma Studies Bank, Iowa City, IA) at 1∶3 dilution; mouse monoclonal anti-human TRA-1-60 and TRA-1-81 (MAB4360 and MAB4381; Chemicon International, Temecula, CA) at 1∶12 and 1∶20 dilutions, respectively; rabbit polyclonal anti-human Nanog and Nestin (ab21624 and ab28944; Abcam Ltd., Cambridge, UK) at 1∶100 dilution; mouse monoclonal anti-Flag (F3165, Sigma) at 1∶1000 dilution. Secondary antibodies conjugated with fluorochromes were purchased from Molecular Probes and used at 1∶400 dilution. Immunofluorescence was performed as previously described [13]. Mouse and human nuclei were distinguished in the resulting heterokaryons by counterstaining with 4,6-diamidino-2-phenylindole (DAPI) and human Lamin A/C staining. Individual cells were delineated by F-actin staining (Phalloidin; A12380, Molecular Probes). For alkaline phosphatase assays, hybrid colonies 8 days after cell fusion were stained with alkaline phosphatase assay kit (Sigma). All slides were analyzed on a Leica TCS SP5 confocal microscope and processed with Leica software and Adobe Photoshop. Images of live GFP fluorescent hybrid colonies and alkaline phosphatase staining were collected using a Leica DM IRE2 microscope running Metamorph software. For FACS analysis a FACScalibur (BD Biosciences) with CellQuest software was used. FACS purification was performed using a FACSAria cell sorter. Western blot analysis was performed as previously described [55] using a goat anti-Oct3/4 polyclonal antibody (sc-8628; Santa Cruz Biotechnology Inc., Santa Cruz, CA) or a mouse anti-Flag monoclonal antibody. As a loading control, blots were incubated with anti-Lamin B polyclonal antibody (sc-6216; Santa Cruz Biotechnology Inc.). Each lane contained 20 ìg total protein.

## Supporting Information

Figure S1 Characterisation of heterokaryon reprogramming of fused hB x mES cells. (A) Human B-lymphocytes (hB) and mouse embryonic stem cells (mES) were respectively labelled with the cell membrane dyes DiI and DiD and fused in the presence of polyethylene glycol (PEG). Fused cells, identified by double-labelling (upper right quadrant), were sorted by FACS and cultured. (B) Mouse and human nuclei were distinguished by FISH using probes specific for mouse γ-satellite DNA (red) or human α-satellite DNA (green), and DAPI counterstained (blue). Confocal sections of human B cells (hB) and mouse ES cells (mES) before and after cell fusion (hB x mES) are shown. Heterokaryons (cells in which parental nuclei share the same cytoplasm but remain discrete, day 1 and 2) were identified up to 2 days after fusion, but by day 3 hybrid formation (where genomes are mixed in the same nucleus, day 3) was detected. Scale bar, 10 µm. (C) Expression of human ES-specific (h*Oct4*, h*Nanog*) and human lymphocyte-specific (h*CD20*, h*CD45*) transcripts detected by RT-PCR using human-specific primers. Prior to fusion, hB cells expressed h*Gapdh*, h*CD20* and h*CD45* but not embryonic stem cell-specific genes. Following heterokaryon formation (hB x mES d2), human pluripotency-associated genes h*Oct4* and h*Nanog* were expressed (upper panel) and h*CD20* and h*CD45* were extinguished (lower panel). mES, -RT and H_2_O were used as negative controls and human embryonic stem cells (hES) as a positive control. h*Gapdh* was used to standardise input. (D) Expression of human h*Tert* transcripts detected by qRT-PCR 0 to 8 days after cell fusion using human-specific primers. Positive (hES-NCL1, black bars) and negative (hB) controls for this analysis were included. Data were normalised to h*Gapdh* expression. Error bars indicate the s.d. of 3 independent experiments.
(5.32 MB TIF)

Figure S2 Differences between human and mouse ES cells and the identification of SSEA4 positive reprogrammed cells. (A) Expression of *Fgfr1*, *Fgfr2*, *Fgf2*, *Bmp4*, *Lifr*, and *Jak3* was assessed by qRT-PCR in human ES cells (hES, NCL1), mouse ES cells (mES) and human B-lymphocytes (hB). *Fgfr1*, *Fgfr2*, and *Fgf2* were uniquely expressed by human ES cells. (B) FACS analysis showed that >90% of hES cells (H1 cell line) expressed SSEA4, while hB and mES do not (2.1% and 1.5% respectively). A proportion of heterokaryons showed SSEA4 expression (15.8%) 8 days after cell fusion (hB x mES d8). (C) FACS sorting of SSEA4 positive cells co-purifies reprogrammed cells that express h*Oct4*, h*Nanog*, and h*Cripto*, as assessed by qRT-PCR. Data were normalised to *Gapdh* expression.
(0.74 MB TIF)

Figure S3 Expression of human-specific embryonic antigens in hybrid cells. Human B cells (hB) and mouse ES cells (mES) were fused and the resulting colonies (hB x mES, day 8) expressed hNanog protein (red) and the human ES-specific antigens SSEA4, TRA-1-81 and TRA-1-60 (green) as assessed by immunofluorescence. Control hB cells did not express any of the markers. DAPI staining is shown in blue. Images are single confocal sections. Scale bar, 50 µm.
(5.14 MB TIF)

Figure S4 Kinetic analysis of Oct4 protein distribution in heterokaryons and the importance of Oct4 for successful reprogramming. (A) Flag-mOct4 ES cells were fused to hB cells and Oct4 protein detected by immunofluorescence at 0, 1, 3, 6, 9, and 12 hours with Oct4 or Flag antibodies (green). Heterokaryons were scored according to the following Oct4 distribution: Oct4 protein not detected (Negative), stronger staining in mES-derived nucleus than hB nucleus (mES>hB), nuclei equally labelled (mES = hB), stronger in the human nucleus (mES<hB). Confocal sections of representative heterokaryons from each of the categories are shown (upper panels). Human nuclei were distinguished from mouse nuclei on basis of diffuse versus punctuate DAPI staining (blue), respectively. Actin labelling (red) delineates the cell membrane. Scale bar, 10 µm. *n* = 100. (B) The ability of mouse ES cells to fuse to human B cells is unaffected by doxycicline (Dox) treatment. ZHBTc4 and hB cells were labelled (with DiD and DiI, respectively) and PEG-fused. Fusion efficiencies were obtained by FACS, as a percentage of double-labelled cells. (C) ZHBTc4 ES cells expressing *Oct4* (black bars), or in which *Oct4* expression has been partially or completely ablated (grey and white bars, respectively) were fused to hB-lymphocytes. The activation of human ES-specific genes (h*Oct4*, h*Nanog*, h*Cripto*, h*Dnmt3b*, h*Sox2*, h*Tle1*, h*Tert*, and h*Rex1*) and silencing of lymphocyte-specific genes (h*CD19*, h*CD45*, and h*CD37*) were quantified by qRT-PCR over the period of 3 days after cell fusion. h*Hprt* was added as a control gene. Data were normalised to h*Gapdh* expression. Error bars indicate the s.d. of 2–3 independent experiments.
(3.61 MB TIF)

Figure S5 siRNA-mediated knock-down of m*Oct4* abolishes reprogramming. (A) E14tg2a ES cells were transfected with either mOct4-siRNA or target-less-siRNA (a negative control siRNA designed to have no expected targets in human and mouse cells) vectors. 48 hours later, transfected cells (GFP+) were FACS sorted and analysed by quantitative RT-PCR analysis. mOct4-siRNA targeted cells showed a >90% reduction in Oct4 transcript levels as compared to cells transfected with target-less-siRNA (control). (B) E14tg2a ES cells expressing mOct4-siRNA or control-siRNA were fused to hB-lymphocytes, and successful reprogramming was assessed by quantifying the abundance of human ES-associated transcripts (h*Nanog* and h*Cripto*) two days after fusion by qRT-PCR. Successful reprogramming judge by the activation of human pluripotency-associated transcripts was abolished by pre-treatment of mES cells with Oct4-siRNAs. Data were normalised to *Gapdh* expression. Error bars indicate the s.d. of 2 independent experiments.
(0.82 MB TIF)

Figure S6 Kinetic of human lymphocyte reprogramming by mES cells after Sox2 ablation. 2TS22C (black bars), Sox2 depleted cells (grey and white bars; Dox 12 and 24 hours, respectively) and 2O1 cells (red bars; Sox2-deficient mES cells in which m*Oct4* expression is constitutively up-regulated) were used as fusion partners with hB cells and reprogramming was assessed by quantification of human-ES transcripts (h*Oct4*, h*Nanog*, h*Cripto*, h*Dnmt3b*, h*Sox2*, h*Tle1*, h*Tert* and h*Rex1*) using qRT-PCR over 3 days after cell fusion. h*Hprt* was added as a control gene. Data were normalised to h*Gapdh* expression. Error bars indicate the s.d. of 2–3 independent experiments.
(0.59 MB TIF)

Figure S7 Characterisation of mouse embryonic hybrid cells. (A) Contribution of the lymphocyte genome within hybrid cells was confirmed by detection of a rearranged *IgH* locus (D–J region). IgH rearrangement was seen in B-lymphocytes (mB), hybrid cells (mES x mB) but not in mES cells. The rearranged DNA can be detected by PCR amplification and visualized on the gel as a 750 bp band. (B) Lymphocyte-specific genes (m*CD19*, m*Pax5*, and m*Ly108*) were not detected in hybrid cells although ES-specific genes (m*Oct4*, m*Nanog*, m*Sox2*, m*Rex1*, and m*Utf1*) remain detectable by RT-PCR. (C) Specific detection of *Oct4* transgene (Oct4βgeo) by RT-PCR with primers within βgeo cassette, which specifically amplify ZHBTc4-derived *Oct4* but not endogenous m*Oct4*. mES and mB cells were included as controls. m*Gapdh* was used to standardise input. (D) Doxycycline (Dox) treatment of ZHBTc4 ES cells results in morphological changes characteristic of trophectoderm differentiation (upper panel). These were not observed in hybrid clones 4 and 12 under the same conditions. GFP protein (Oct4 promoter-driven) remains detectable in hybrid cells throughout the experiment, as assessed by immunofluorescence.
(7.86 MB TIF)

Table S1 Primers used in this study.
(0.17 MB DOC)

Text S1 Supplementary methods.
(0.04 MB DOC)
