# Supplementary figures and images for: Heterokaryon-Based Reprogramming of Human B Lymphocytes for Pluripotency Requires Oct4 but Not Sox2
Source: PLoS Genet. 2008 Sep 5;4(9):e1000170. doi: 10.1371/journal.pgen.1000170 (PMC2527997; doi:10.1371/journal.pgen.1000170)

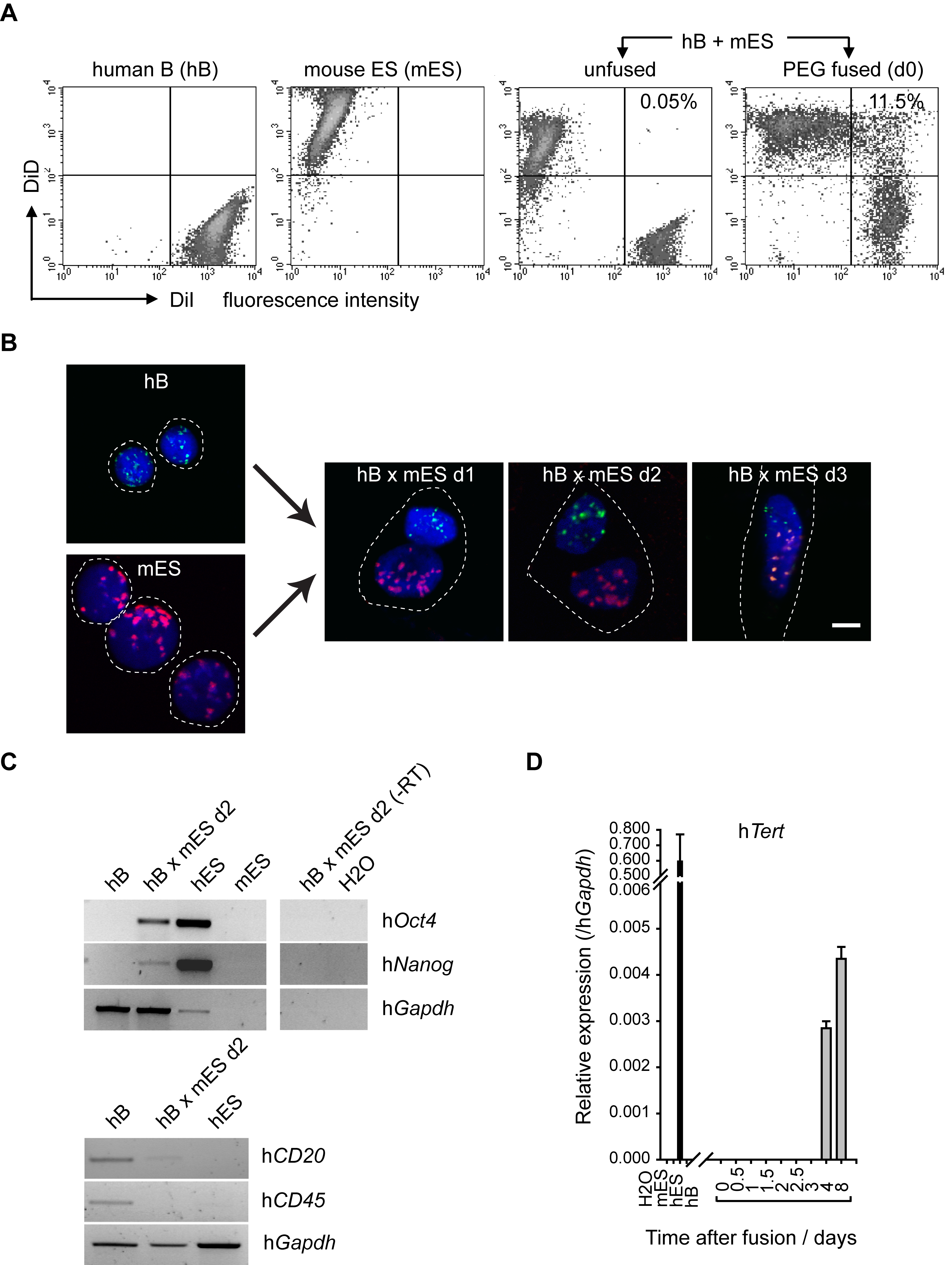

Supplement: Figure S1 — Characterisation of heterokaryon reprogramming of fused hB x mES cells. (A) Human B-lymphocytes (hB) and mouse embryonic stem cells (mES) were respectively labelled with the cell membrane dyes DiI and DiD and fused in the presence of polyethylene glycol (PEG). Fused cells, identified by double-labelling (upper right quadrant), were sorted by FACS and cultured. (B) Mouse and human nuclei were distinguished by FISH using probes specific for mouse γ-satellite DNA (red) or human α-satellite DNA (green), and DAPI counterstained (blue). Confocal sections of human B cells (hB) and mouse ES cells (mES) before and after cell fusion (hB x mES) are shown. Heterokaryons (cells in which parental nuclei share the same cytoplasm but remain discrete, day 1 and 2) were identified up to 2 days after fusion, but by day 3 hybrid formation (where genomes are mixed in the same nucleus, day 3) was detected. Scale bar, 10 µm. (C) Expression of human ES-specific (hOct4, hNanog) and human lymphocyte-specific (hCD20, hCD45) transcripts detected by RT-PCR using human-specific primers. Prior to fusion, hB cells expressed hGapdh, hCD20 and hCD45 but not embryonic stem cell-specific genes. Following heterokaryon formation (hB x mES d2), human pluripotency-associated genes hOct4 and hNanog were expressed (upper panel) and hCD20 and hCD45 were extinguished (lower panel). mES, -RT and H2O were used as negative controls and human embryonic stem cells (hES) as a positive control. hGapdh was used to standardise input. (D) Expression of human hTert transcripts detected by qRT-PCR 0 to 8 days after cell fusion using human-specific primers. Positive (hES-NCL1, black bars) and negative (hB) controls for this analysis were included. Data were normalised to hGapdh expression. Error bars indicate the s.d. of 3 independent experiments. (5.32 MB TIF) [file pgen.1000170.s001.tif]

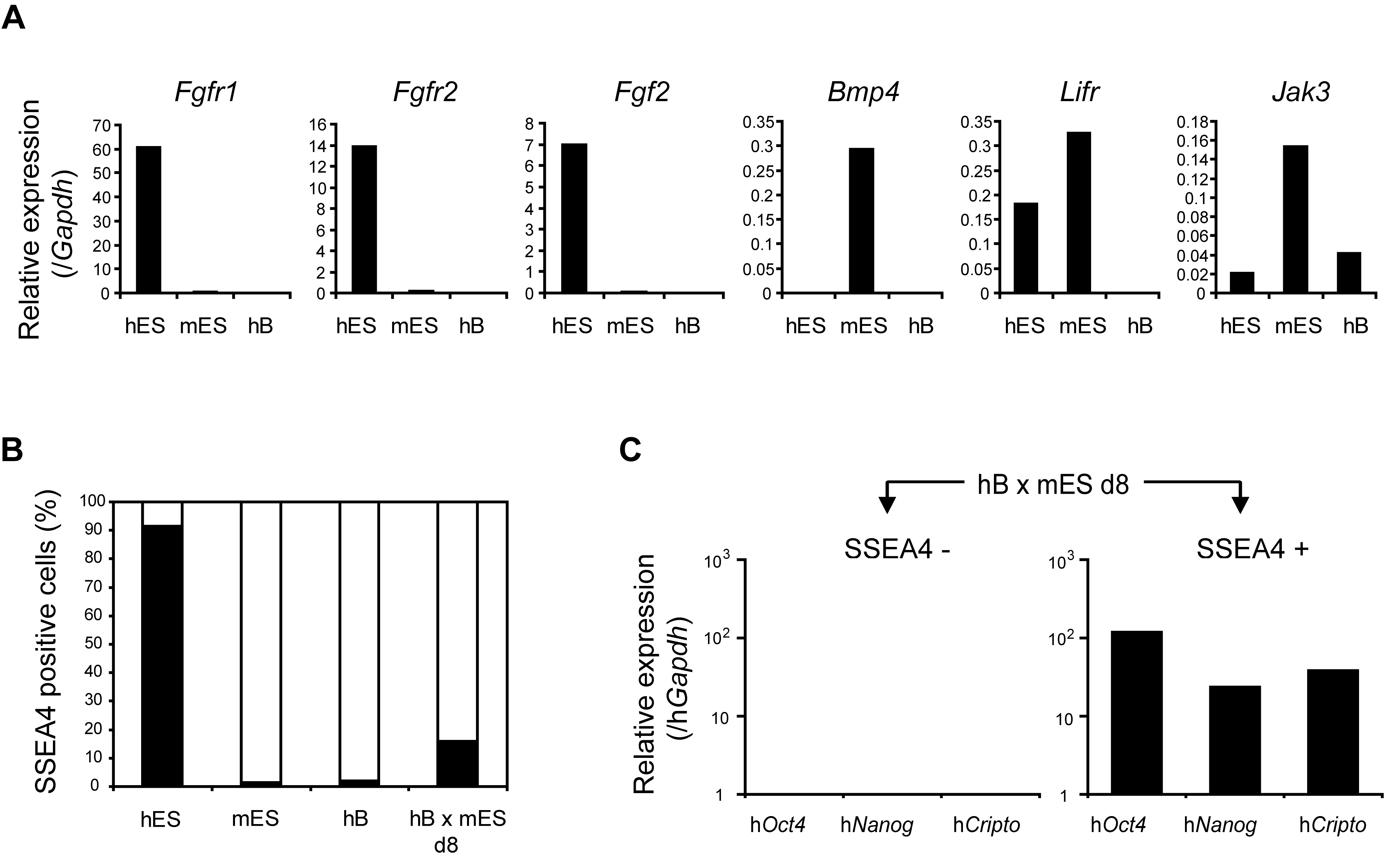

Supplement: Figure S2 — Differences between human and mouse ES cells and the identification of SSEA4 positive reprogrammed cells. (A) Expression of Fgfr1, Fgfr2, Fgf2, Bmp4, Lifr, and Jak3 was assessed by qRT-PCR in human ES cells (hES, NCL1), mouse ES cells (mES) and human B-lymphocytes (hB). Fgfr1, Fgfr2, and Fgf2 were uniquely expressed by human ES cells. (B) FACS analysis showed that >90% of hES cells (H1 cell line) expressed SSEA4, while hB and mES do not (2.1% and 1.5% respectively). A proportion of heterokaryons showed SSEA4 expression (15.8%) 8 days after cell fusion (hB x mES d8). (C) FACS sorting of SSEA4 positive cells co-purifies reprogrammed cells that express hOct4, hNanog, and hCripto, as assessed by qRT-PCR. Data were normalised to Gapdh expression. (0.74 MB TIF) [file pgen.1000170.s002.tif]

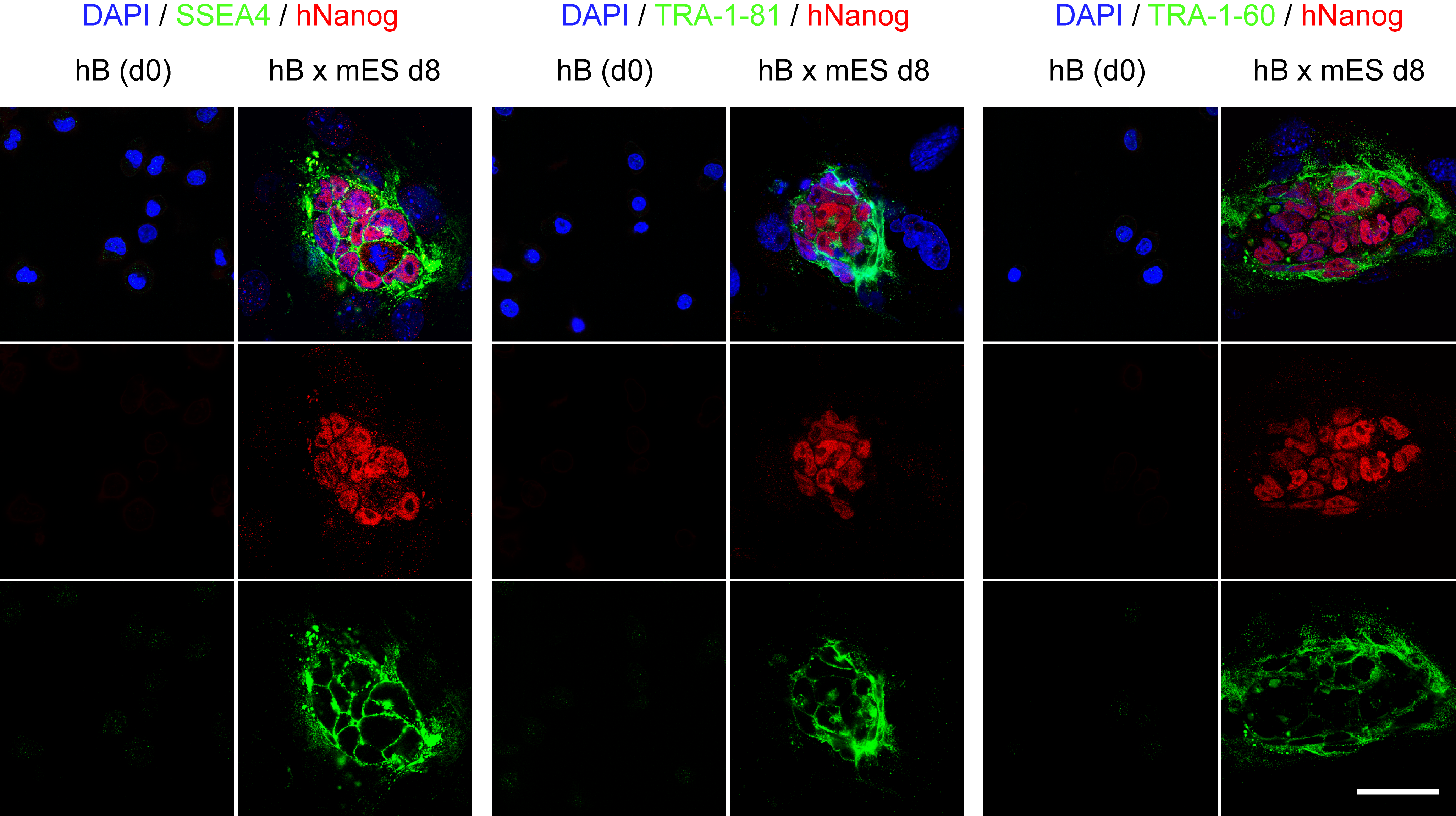

Supplement: Figure S3 — Expression of human-specific embryonic antigens in hybrid cells. Human B cells (hB) and mouse ES cells (mES) were fused and the resulting colonies (hB x mES, day 8) expressed hNanog protein (red) and the human ES-specific antigens SSEA4, TRA-1-81 and TRA-1-60 (green) as assessed by immunofluorescence. Control hB cells did not express any of the markers. DAPI staining is shown in blue. Images are single confocal sections. Scale bar, 50 µm. (5.14 MB TIF) [file pgen.1000170.s003.tif]

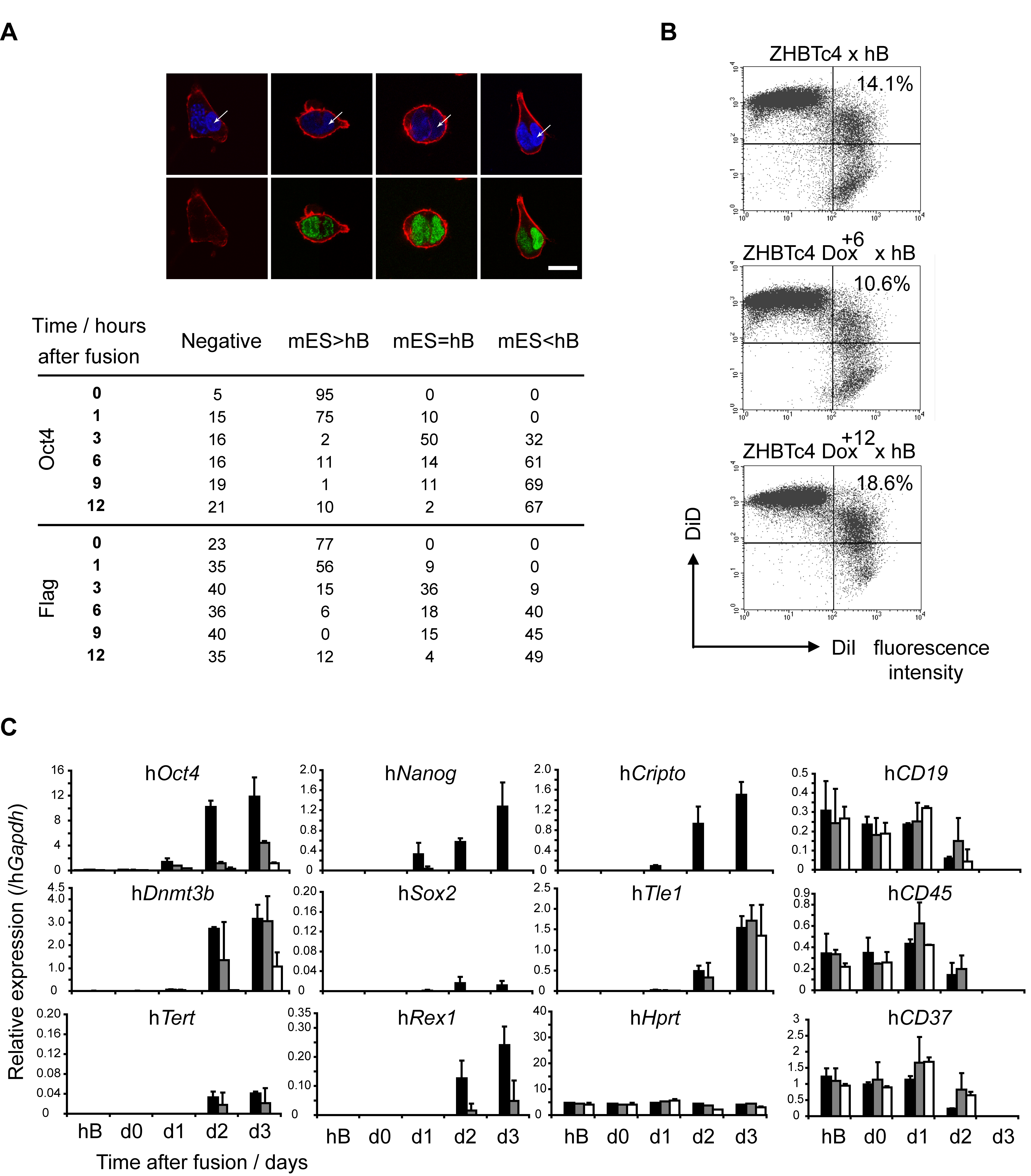

Supplement: Figure S4 — Kinetic analysis of Oct4 protein distribution in heterokaryons and the importance of Oct4 for successful reprogramming. (A) Flag-mOct4 ES cells were fused to hB cells and Oct4 protein detected by immunofluorescence at 0, 1, 3, 6, 9, and 12 hours with Oct4 or Flag antibodies (green). Heterokaryons were scored according to the following Oct4 distribution: Oct4 protein not detected (Negative), stronger staining in mES-derived nucleus than hB nucleus (mES>hB), nuclei equally labelled (mES = hB), stronger in the human nucleus (mES<hB). Confocal sections of representative heterokaryons from each of the categories are shown (upper panels). Human nuclei were distinguished from mouse nuclei on basis of diffuse versus punctuate DAPI staining (blue), respectively. Actin labelling (red) delineates the cell membrane. Scale bar, 10 µm. n = 100. (B) The ability of mouse ES cells to fuse to human B cells is unaffected by doxycicline (Dox) treatment. ZHBTc4 and hB cells were labelled (with DiD and DiI, respectively) and PEG-fused. Fusion efficiencies were obtained by FACS, as a percentage of double-labelled cells. (C) ZHBTc4 ES cells expressing Oct4 (black bars), or in which Oct4 expression has been partially or completely ablated (grey and white bars, respectively) were fused to hB-lymphocytes. The activation of human ES-specific genes (hOct4, hNanog, hCripto, hDnmt3b, hSox2, hTle1, hTert, and hRex1) and silencing of lymphocyte-specific genes (hCD19, hCD45, and hCD37) were quantified by qRT-PCR over the period of 3 days after cell fusion. hHprt was added as a control gene. Data were normalised to hGapdh expression. Error bars indicate the s.d. of 2–3 independent experiments. (3.61 MB TIF) [file pgen.1000170.s004.tif]

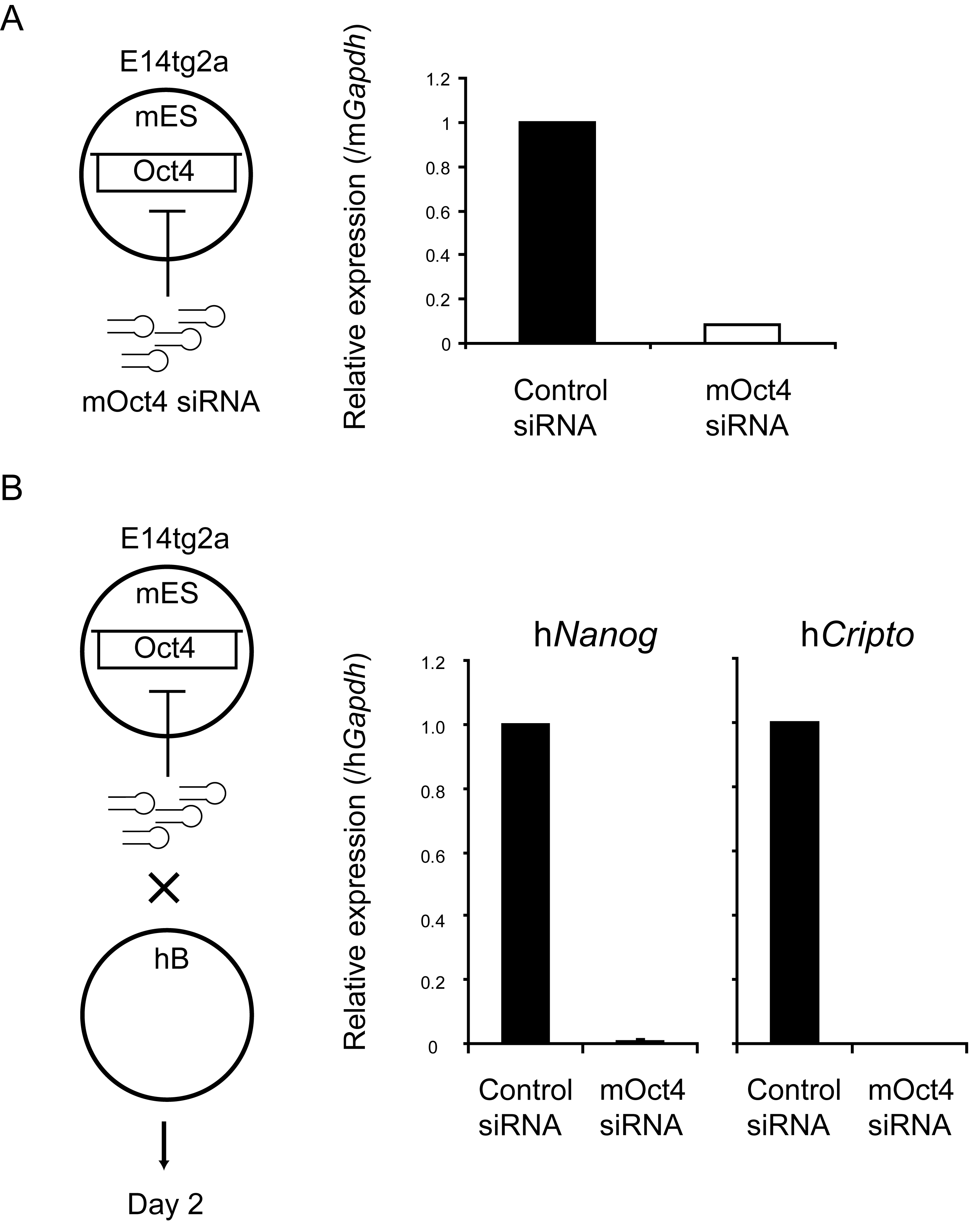

Supplement: Figure S5 — siRNA-mediated knock-down of mOct4 abolishes reprogramming. (A) E14tg2a ES cells were transfected with either mOct4-siRNA or target-less-siRNA (a negative control siRNA designed to have no expected targets in human and mouse cells) vectors. 48 hours later, transfected cells (GFP+) were FACS sorted and analysed by quantitative RT-PCR analysis. mOct4-siRNA targeted cells showed a >90% reduction in Oct4 transcript levels as compared to cells transfected with target-less-siRNA (control). (B) E14tg2a ES cells expressing mOct4-siRNA or control-siRNA were fused to hB-lymphocytes, and successful reprogramming was assessed by quantifying the abundance of human ES-associated transcripts (hNanog and hCripto) two days after fusion by qRT-PCR. Successful reprogramming judge by the activation of human pluripotency-associated transcripts was abolished by pre-treatment of mES cells with Oct4-siRNAs. Data were normalised to Gapdh expression. Error bars indicate the s.d. of 2 independent experiments. (0.82 MB TIF) [file pgen.1000170.s005.tif]

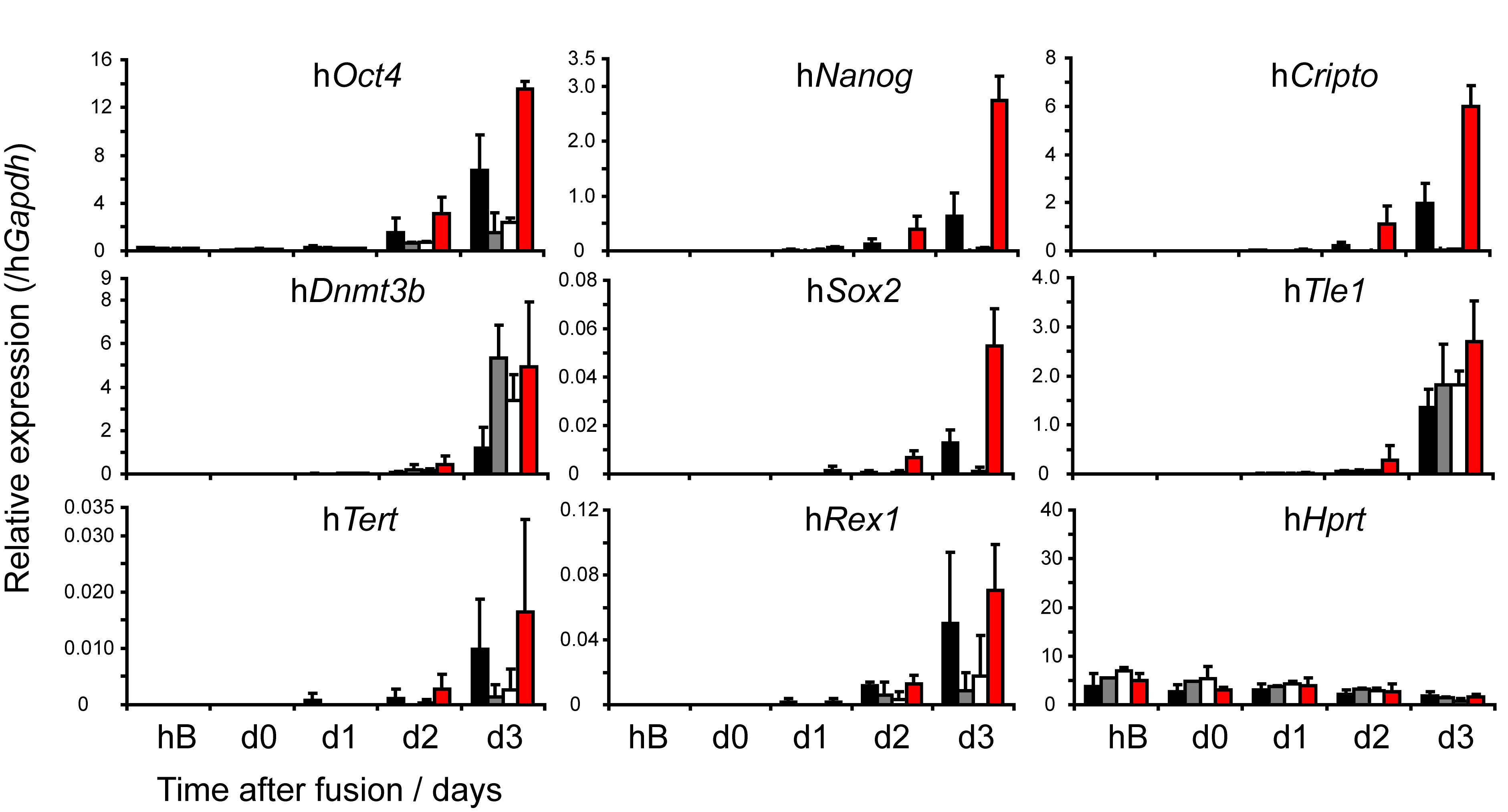

Supplement: Figure S6 — Kinetic of human lymphocyte reprogramming by mES cells after Sox2 ablation. 2TS22C (black bars), Sox2 depleted cells (grey and white bars; Dox 12 and 24 hours, respectively) and 2O1 cells (red bars; Sox2-deficient mES cells in which mOct4 expression is constitutively up-regulated) were used as fusion partners with hB cells and reprogramming was assessed by quantification of human-ES transcripts (hOct4, hNanog, hCripto, hDnmt3b, hSox2, hTle1, hTert and hRex1) using qRT-PCR over 3 days after cell fusion. hHprt was added as a control gene. Data were normalised to hGapdh expression. Error bars indicate the s.d. of 2–3 independent experiments. (0.59 MB TIF) [file pgen.1000170.s006.tif]

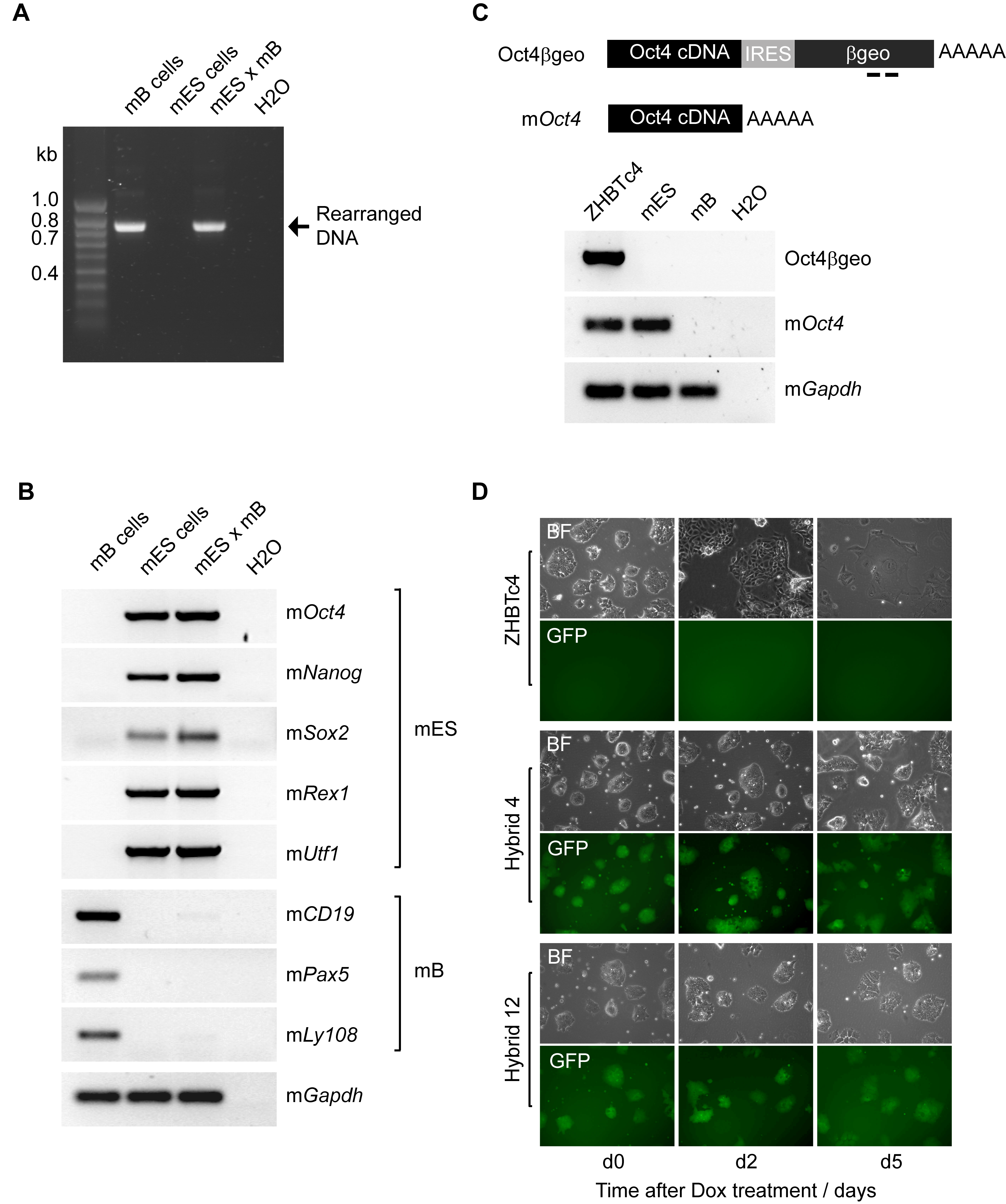

Supplement: Figure S7 — Characterisation of mouse embryonic hybrid cells. (A) Contribution of the lymphocyte genome within hybrid cells was confirmed by detection of a rearranged IgH locus (D–J region). IgH rearrangement was seen in B-lymphocytes (mB), hybrid cells (mES x mB) but not in mES cells. The rearranged DNA can be detected by PCR amplification and visualized on the gel as a 750 bp band. (B) Lymphocyte-specific genes (mCD19, mPax5, and mLy108) were not detected in hybrid cells although ES-specific genes (mOct4, mNanog, mSox2, mRex1, and mUtf1) remain detectable by RT-PCR. (C) Specific detection of Oct4 transgene (Oct4βgeo) by RT-PCR with primers within βgeo cassette, which specifically amplify ZHBTc4-derived Oct4 but not endogenous mOct4. mES and mB cells were included as controls. mGapdh was used to standardise input. (D) Doxycycline (Dox) treatment of ZHBTc4 ES cells results in morphological changes characteristic of trophectoderm differentiation (upper panel). These were not observed in hybrid clones 4 and 12 under the same conditions. GFP protein (Oct4 promoter-driven) remains detectable in hybrid cells throughout the experiment, as assessed by immunofluorescence. (7.86 MB TIF) [file pgen.1000170.s007.tif]
