# Supplementary material for: Heterokaryon-Based Reprogramming of Human B Lymphocytes for Pluripotency Requires Oct4 but Not Sox2
Source: PLoS Genet. 2008 Sep 5;4(9):e1000170. doi: 10.1371/journal.pgen.1000170 (PMC2527997; doi:10.1371/journal.pgen.1000170)
Supplement: Table S1 — Primers used in this study. (0.17 MB DOC) [file pgen.1000170.s008.doc]

Table S1. Primers used in this study

| Human specific primers for qRT-PCR | | | |
| --- | --- | --- | --- |
| *Species/Gene* | *Accession number* |  | *Sequence 5’-3’* |
| h*Gapdh* | NM_002046 | s | TCTGCTCCTCCTGTTCGACA |
|  |  | as | AAAAGCAGCCCTGGTGACC |
| h*Hprt* | NM_000194 | s | TCCTTGGTCAGGCAGTATAATCC |
|  |  | as | GTCAAGGGCATATCCTACAACAAA |
| h*Oct4* | NM_002701 | s | TCGAGAACCGAGTGAGAGGC |
|  |  | as | CACACTCGGACCACATCCTTC |
| h*Nanog* | NM_024865 | s | CCAACATCCTGAACCTCAGCTAC |
|  |  | as | GCCTTCTGCGTCACACCATT |
| h*Cripto* | NM_003212 | s | AGAAGTGTTCCCTGTGTAAATGCTG |
|  |  | as | CACGAGGTGCTCATCCATCA |
| h*Dnmt3b* | NM_006892 | s | GTCAAGCTACACACAGGACTTGACAG |
|  |  | as | AGTTCGGACAGCTGGGCTTT |
| h*Tert* | NM_198253 | s | GCCAGCATCATCAAACCCC |
|  |  | as | CTGTCAAGGTAGAGACGTGGCTC |
| h*Tle1* | NM_005077 | s | TGTCTCCCAGCTCGACTGTCT |
|  |  | as | AAGTACTGGCTTCCCCTCCC |
| h*Sox2* | NM_003106 | s | cacactgcccctctcacacat |
|  |  | as | catttccctcgtttttctttgaa |
| h*Rex1* | NM_174900 | s | GCGTACGCAAATTAAAGTCCAGA |
|  |  | as | CAGCATCCTAAACAGCTCGCAGAAT |
| h*CD37* | NM_001774 | s | GTGGCTGCACAACAACCTTATTT |
|  |  | as | GCCTAACGGTATCGAGCGAG |
| h*CD19* | NM_001770 | s | GCTCAAGACGCTGGAAAGTATTATT |
|  |  | as | GATAAGCCAAAGTCACAGCTGAGA |
| h*CD20* | NM_152866 | s | CCCCATCTACCCAATACTGTTACAG |
|  |  | as | TGGGTCTGGAGCACGTTCTT |
| h*CD45* | NM_002838 | s | CCCCATGAACGTTACCATTTG |
|  |  | as | GATAGTCTCCATTGTGAAAATAGGCC |
| h*Pax5* | NM_016734 | s | AGCTTCCAGTCACAGCATAGTGTC |
|  |  | as | ACCTTCGTCTCTCTTGCGCTT |
| h*Fgfr1* | NM_023105 | s | GCAAGTGAGAGCTTCCTGAGCT |
|  |  | as | ACCTTCAATCGAGGCAGGAA |
| h*Fgfr2* | NM_000141 | s | GCCGCCGTGATCAGTTG |
|  |  | as | GCGCCCTTTATCTGCAAGTACT |
| h*Fgf2* | NM_002006 | s | TCACATCAAGCTACAACTTCAAGCA |
|  |  | as | AAGCCAGTAATCTTCCATCTTCCTT |
| h*Bmp4* | NM_001202 | s | TGGTCTTGAGTATCCTGAGCG |
|  |  | as | GCTGAGGTTAAAGAGGAAACGA |
| h*Lifr* | NM_002310 | s | CCAACATGACTTGCGACTACGT |
|  |  | as | CCTGGTCGAAACTCATCAGATTCTAT |
| h*Jak3* | NM_000215 | s | AAGCAGCGAGCTTGATGAGC |
|  |  | as | GTCTATGGCCCCCAGGTGTA |
| h*ALPL* | NM_000478 | s | CCTGGCAGGGCTCACACT |
|  |  | as | AAACAGGAGAGTCGCTTCAGAGA |
| h*CollagenIVα1* | NM_001845 | s | TCAGCAGGGCATCGCAT |
|  |  | as | AAATGTCATTTCAGGCCTAGTGG |
| h*Nestin* | NM_006617 | s | TGTGGCCCAGAGGCTTCTC |
|  |  | as | CAGGGCTGGTGAGCTTGG |
| h*Hnf4* | NM_175914 | s | ACCAGCTACATCGCCTACCTGA |
|  |  | as | CAGGAGGAAAACCTTCGTGCT |
| h*Mixl1* | NM_031944 | s | CCGAGTCCAGGATCCAGGTA |
|  |  | as | CTCTGACGCCGAGACTTGG |
| h*Sox7* | NM_031439 | s | ACGCCGAGCTCAGCAAGAT |
|  |  | as | TCCACGTACGGCCTCTTCTG |
| h*Gata6* | NM_005257 | s | ACCACCTTATGGCGCAGAAAC |
|  |  | as | TTTTTCATAGCAAGTGGTCTGGG |
| h*Cdx2* | NM_001265 | s | CAGGACGAAAGACAAATATCGAGTG |
|  |  | as | CCAGATTTTAACCTGCCTCTCAGA |
| h*Hand1* | NM_004821 | s | ACCAGCTACATCGCCTACCTGA |
|  |  | as | CAGGAGGAAAACCTTCGTGCT |
| h*Ebf* | NM_024007 | s | GGAGATCGAGAGGACAGCGT |
|  |  | as | GTCAATGAGGCGCACGTAGA |
| h*MyoD* | NM_002478 | s | AACTTAAATGCCCCCCTCCCC |
|  |  | as | TTCAGTTCTCCGCCTCTCCTAC |
| Mouse primers for qRT-PCR | | | |
| *Species/Gene* | *Accession number* |  | *Sequence 5’-3’* |
| m*Gapdh* | NM_008084 | s | TGCACCACCAACTGCTTAGC |
|  |  | as | GGCATGGACTGTGGTCATGAG |
| m*Oct4* | NM_013633 | s | CGTGGAGACTTTGCAGCCTG |
|  |  | as | GCTTGGCAAACTGTTCTAGCTCCT |
| m*Nanog* | NM_028016 | s | GAACTATTCTTGCTTACAAGGGTCTGC |
|  |  | as | GCATCTTCTGCTTCCTGGCAA |
| m*Cripto* | NM_011562 | s | CACCAACCCAGGGTATCAGTT |
|  |  | as | AGAGTTCTGTCCAGTGTCGTC |
| m*Dnmt3b* | NM_001003961 | s | ACTGCCTGGAGTTCAGTAGGA |
|  |  | as | CCCTGTCTGATGGAGTTCGAC |
| m*Sox2* | NM_011443 | s | GAGTGGAAACTTTTGTCCGAGA |
|  |  | as | GAAGCGTGTACTTATCCTTCTTCAT |
| m*Tle1* | NM_011599 | s | TCTGAGTGGGCAGTCTCACTT |
|  |  | as | GAAGAAGGGTCCTCGTTAGACA |
| m*Tert* | NM_009354 | s | TCAAGAGCAGTAGTCGCCAG |
|  |  | as | TCTCGGGACAGGATAGCATCT |
| m*Rex1* | NM_009556 | s | CTCCTAGCCGCCTAGATTTCCA |
|  |  | as | CGTGTCCCAGCTCTTAGTCCATT |
| m*Fgfr1* | NM_010206 | s | GCAGAGCATCAACTGGCTG |
|  |  | as | GGTCACGCAAGCGTAGAGG |
| m*Fgfr2* | NM_010207 | s | GAGGAATACTTGGATCTCACCCAGC |
|  |  | as | CTGGTGCTGTCCTGTTTGGG |
| m*Fgf2* | NM_008006 | s | GGCTCTACTGCAAGAACGGC |
|  |  | as | TGGAGTTGTAGTTTGACGTGTGG |
| m*Bmp4* | NM_007554 | s | GAGGGATCTTTACCGGCTCC |
|  |  | as | GTTGAAGAGGAAACGAAAAGCAG |
| m*Lifr* | NM_013584 | s | GCAGAGCATCAACTGGCTG |
|  |  | as | GGTCACGCAAGCGTAGAGG |
| m*Jak3* | NM_010589 | s | CTTCCGCCTGATCTGCGAC |
|  |  | as | TGGCAAAGTCTAACGTGATGG |
| m*Cdx2* | NM_007673 | s | TCAACCTCGCCACAACCTTCCC |
|  |  | as | TGGCTCAGCCTGGGATTGCT |
| m*Hand1* | NM_008213 | s | ACGTGCTGGCCAAGGATGCA |
|  |  | as | TGGTTTAGCTCCAGCGCCCA |
| m*CD19* | NM_009844 | s | GGAGAGCACCCGGTCAGA |
|  |  | as | CCACACTGCTGACCTTGCAA |
| m*CD37* | NM_007645 | s | AAGTACTTCCTCTTCGTTTTCAACCT |
|  |  | as | ACCTGAGACAGCCAGGACCTT |
| m*CD45* | NM_011210 | s | TGTACCACCAGGGACTGACAAG |
|  |  | as | TCTGGCTCACAGTGGAGTACATATG |
| Oct4βgeo transgene (within neo^r^ gene) |  | s | CGGCAGGAGCAAGGTGAGAT |
|  |  | as | CAAGATGGATTGCACGCAGG |
| Bisulfite genomic sequencing | | | |
| BGS for h*Oct4* region 1 |  | s | TAGTTGGGATGTGTAGAGTTTGAGA |
|  |  | as | TAAACCAAAACAATCCTTCTACTCC |
| BGS for h*Oct4* region 2 |  | s | AAGTTTTTGTGGGGGATTTGTAT |
|  |  | as | CCACCCACTAACCTTAACCTCTA |
| BGS for h*Oct4* region 3 |  | s | GTTAGAGGTTAAGGTTAGTGGGTG |
|  |  | as | AAACCTTAAAAACTTAACCAAATCC |
| BGS for h*Igf2/hH19* ICR |  | s | TGTTGAAGGTTGGGGAGATGGGA |
|  |  | as | CCCAAACCATAACACTAAAACCCTC |
| Genomic PCR | | | |
| *IgH* locus (D-J region) |  | s | TTCAAAGCACAATGCCTGGCT |
|  |  | as | GTCTAGATTCTCACAAGAGTCCGATAGACCCTGG |
