# Supplementary material for: Heterokaryon-Based Reprogramming of Human B Lymphocytes for Pluripotency Requires Oct4 but Not Sox2
Source: PLoS Genet. 2008 Sep 5;4(9):e1000170. doi: 10.1371/journal.pgen.1000170 (PMC2527997; doi:10.1371/journal.pgen.1000170)
Supplement: Text S1 — Supplementary methods. (0.04 MB DOC) [file pgen.1000170.s009.doc]

**SUPPLEMENTARY METHODS**

**Fluorescence *in situ* hybridization (FISH)**

Mouse γ-satellite (major-satellite) probe was directly labelled with Fluoro-RED and used in combination with DIG-coupled human α-satellite probe (Q-Biogen, Illkirch, France). α-satellite was detected with anti-digoxygenin fluorescein isothiocyanate (AD-FITC) raised in sheep (Boehringer, Mannheim, Germany), followed by anti-sheep FITC (Vector Laboratories Inc.). Cells were harvested by trypsinisation, and hypotonically treated in 75 mM KCl for 5min at room temperature before fixation in ice-cold methanol: acetic acid (3:1). Slides were denatured for 2.5 min at 75 °C in 70% formamide and 2xSSCP, immediately passed through an ice-cold ethanol series (70, 90 and 100%) and allowed to air-dry. The probes were resuspended in 15 μl hybridization solution, denatured at 75 °C for 3 min, pre-annealed at 37 °C and then hybridized overnight at 37 °C. Slides were washed as follows: 50% formamide and 2xSSC (45 °C, 3x5 min); 0.5xSSC (60 °C, 3x5 min); SSCT, (RT, 1x5 min).

**Mouse *Oct4* expression knock down by siRNA**

Oligonucleotides for RNA interference were designed using available on-line software (<http://jura.wi.mit.edu/bioc/siRNAext/home.php>). siRNA sequences used for mouse Oct4 downregulation (and not human) were 5′-GATCCCCGAAGGATGTGGTTCGAGTATTCAAGAGATACTCGAACCACATCCTTCTTTTTA-3’ and 5′-AGCTTAAAAAGAAGGATGTGGTTCGAGTATCTCTTGAATACTCGAACCACATCCTTCGGG-3’. A short hairpin with no expected targets in both human and mouse cells was included as a control: 5’-GATCCCCGcgcgctttgtaggattcgTTCAAGAGAcgaatcctacaaagcgcgcTTTTTA-3’ and 5’-AGCTTAAAAAGcgcgctttgtaggattcgTCTCTTGAAcgaatcctacaaagcgcgcGGG-3’. The hairpins were synthesised, HPLC purified (Sigma), annealed and cloned into pSUPER.Neo+GFP vector (VEC-PBS-0005; OligoEngine, Inc, Seattle, WA). The expression of the siRNA in this vector is driven by the PolIII dependent H1 promoter and the selection markers (Neo^r^ and EGFP) are driven by the PGK promoter. The vectors were transfected in E14tg2a ES cells using lipofectamine 2000 (Invitrogen) and 48 hours later, transfected cells (GFP-positive) were FACS sorted and used for cell fusion experiments.

**Genomic PCR**

Genomic DNA was isolated using phenol-chlorophorm extraction (Sigma). Presence of rearranged DNA of the *IgH* locus (D-J region) was checked by PCR using HotStarTaq^TM^ (Qiagen) DNA polymerase (35 cycles of 94°C for 30 sec; 60°C for 30 sec and 72°C for 1 min) with primers found in Table S1.
